# Integrated Chemical and Toxicity Screening of Tap Drinking Water across Western Oregon Using Suspect and Nontarget Screening

**DOI:** 10.1021/acs.est.6c01661

**Published:** 2026-06-17

**Authors:** Peter W. Bright, Miranda E. Jackson, Chloe L. Fender, Kenneth Lee, Caoilinn Haggerty, Jason Schindler, Lisa Truong, Robyn L. Tanguay, Manuel Garcia-Jaramillo

**Affiliations:** † Department of Environmental and Molecular Toxicology, 508120 Oregon State University, Corvallis, Oregon 97330, United States; ‡ Sinnhuber Aquatic Research Laboratory, Oregon State University, Corvallis, Oregon 97333, United States

**Keywords:** prioritization, zebrafish toxicity screening, early life toxicity screening, sublethal toxicity screening, contaminant mixtures, mass spectrometry, drinking water contaminants

## Abstract

Drinking water contamination poses ongoing public health concerns due to limited analytical and toxicological coverage of emerging and unknown chemical contaminants. Here, we integrate suspect screening and nontarget screening with *in vivo* toxicity testing to characterize organic chemical contaminants in tap drinking water across western Oregon, USA. Twelve public tap drinking water samples and one commercially bottled water sample were analyzed using complementary extraction and chromatographic workflows paired with high-resolution mass spectrometry analysis. Suspect screening identified 52 Level 1–2 annotated compounds, with prioritization leading to 13 chemical confirmations including both known drinking water contaminants (e.g., propiconazole, benzyl butyl phthalate, saccharin) and previously unreported drinking water contaminants (e.g., 18β-glycyrrhetinic acid). Zebrafish embryo and larvae toxicity screening demonstrated significant morphological toxicity following exposure to 18β-glycyrrhetinic acid and a benzyl butyl phthalate/monobenzyl phthalate mixture, while behavioral assays identified altered behavior following exposure to one individual contaminant and seven mixtures. This integrated analytical–toxicological framework demonstrates broad physicochemical screening, expands the inventory of confirmed drinking water contaminants, and establishes primary reference concentration values at the 20% effect level for one novel drinking water contaminant and several representative contaminant mixtures.

## Graphical abstract

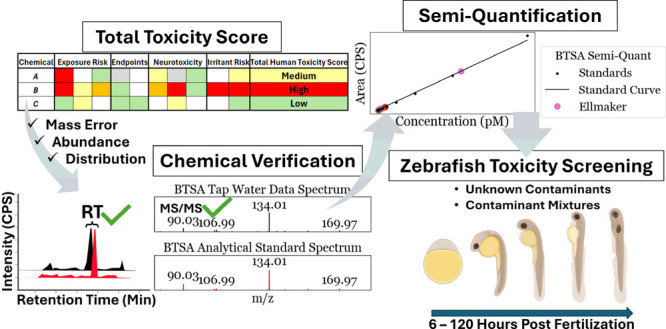

## 1 Introduction

Safeguarding drinking water quality remains a critical challenge, as continued synthesis, production, and use of existing and emerging chemicals inevitably alters the composition of public drinking water sources.^1,2^ Globally, an estimated two billion people rely on drinking water sources that do not meet quality standards.^3–5^ In the United States approximately 90% of the population relies on tap water provided by public water systems as their primary source of drinking water.^6^ With 290 million entries in the Chemical Abstracts Service Registry and more than 86,000 chemicals listed under the U.S. Toxic Substances Control Act inventory,^7,8^ contaminated drinking water poses a risk of chemical exposure to millions of individuals.

Correlating human health outcomes with the consumption of contaminated drinking water remains challenging. Many contaminants occur at concentrations below conventional thresholds of concern, and exposure can occur over chronic time scales.^9,10^ Moreover, the chemical composition of tap water is complex and variable. Increasing pressures of climate change, population growth, and continued chemical synthesis and production encourage use of alternative sources of drinking water, such as potable reuse, where chemical contamination is expected to be regionally varied based on industrialization, urbanization, and environmental conditions.^11^ Routine regulatory assessments of drinking water focus on known contaminants, potentially overlooking unknown or emerging contaminants.^1,2,4^ Broader chemical screening methods often rely on extraction, preconcentration, or isolation procedures that inherently target specific physicochemical properties.^4,12–14^ Despite analytical constraints, mounting evidence suggests chronic exposure to contaminated drinking water contributes to impaired neurological and developmental effects.^15^ Such exposures are particularly concerning during early developmental stages, when disruptions to the formation of the central nervous system may lead to increased susceptibility to chronic disease later in life.^9,10^ Due to ethical and practical limitations of studying developmental effects directly in humans, vertebrate models are essential for assessing the impacts of complex chemical exposures. Zebrafish (*Danio rerio*) are a relatively rapid and cost-effective *in vivo* vertebrate model, enabling high-throughput behavioral and neurobiological research with translational relevance to human biology.^16,17^ Bioactivity in zebrafish frequently predicts effects in rodents and humans, and many chemicals that affect human development or the central nervous system elicit comparable responses in zebrafish.^18^ Consequently, zebrafish models are widely used for neurophenotyping, genetic studies, drug screening, and the investigation of complex neurological and psychiatric disorders.^19^

High-resolution mass spectrometry (HRMS) combined with suspect screening (SS) and nontarget screening (NTS) has emerged as a powerful approach for broad characterization of organic contaminants in drinking water.^4,13,14,20^ High-resolution instruments such as quadrupole time-of-flight (QToF) mass spectrometers enable resolution of thousands of spectral features without requiring predefined targets.^21^ Preconcentration and chromatography techniques are readily integrated to improve method sensitivity for trace contaminants in drinking water.^1^ Further, a diverse range of physicochemical contaminants may be targeted with different methods, such as the use of hydrophilic interaction chromatography (HILIC) to target highly polar contaminants that are poorly retained in solely reverse phase (RP) methods.^13,22–24^ Nevertheless, between 2013 and 2024 fewer than 175 unique drinking water contaminants were confirmed at the Level 1 confidence interval, and few studies linked results directly to human health outcomes.^4^ These findings highlight a need for integrated approaches, where following broad contaminant identification, meaningful toxicity evaluation can be performed.

In this study, we present an integrated analytical-toxicological framework for broad organic contaminant screening of tap drinking waters with samples collected from 12 public water systems across western Oregon and one commercially bottled water source (*n* = 13). Analyses were intended to screen tap water for unregulated and unknown contaminants and to assess their toxicity using the well-established zebrafish developmental toxicity assay.^25^ A bimodal extraction and chromatographic workflow utilizing solid-phase extraction (SPE) coupled with RP chromatography and nitrogen evaporative preconcentration (N_2_ evap) coupled with HILIC was applied to broaden the physicochemical coverage of the analysis. Thirteen chemical contaminant annotations were confirmed through prioritization, and eight chemicals and 13 contaminant mixtures were evaluated for toxicity using zebrafish embryo and larvae assays.

## 2 Materials and Methods

### 2.1 Sample Collection

Tap drinking water was collected during two sampling events on October 28th and 30th, 2022, from 12 different public water systems across western Oregon and one bottled and commercially purified source (Figure S1). Samples were collected from the same water source twice roughly 48 h apart, with the exception of 72 h for the bottled water. Water samples were chosen to cover a variety of source water types (surface water, groundwater, or mixed), municipal treatment processes (highly treated to chlorination only), and community sizes served across western Oregon. Water source information was gathered from Oregon Health Authority’s Oregon Public Water Systems Web site^26^ and is presented in Table S1.

Water samples were collected directly from tap water sources into precleaned, methanol rinsed 1000 mL polyethylene bottles. Tap water sources were allowed to run for 30 s, then bottles were rinsed three times before being filled completely and capped tightly. Samples were immediately transferred to a cooler with ice for transport back to Oregon State University and stored at 4 °C. Following the second sampling event duplicate water samples were combined and mixed to control for short-term temporal variability, and were extracted for each site within 72 h. A field blank was prepared for each sampling day by collecting milli-Q water (18.2 MΩ cm) into precleaned bottles, which were then combined and extracted following the same protocol used for the tap water samples.

### 2.2 Sample Extraction

Two individual extraction protocols were applied to each water source sample to target different portions of the chemical constituency (Figure S2a). Full extraction details are provided in the Supporting Information (SI). The SPE extraction method resulted in a 1 mL extract from a starting 1000 mL sample while N_2_ evap resulted in a 400 μL extract from a starting 10 mL sample aliquot, resulting in final sample preconcentration factors of 1000x and 25x, respectively.

### 2.3 LC-HRMS Analysis

Water extracts were analyzed using liquid-chromatography coupled to high-resolution mass spectrometry (LC-HRMS). Water extracts were analyzed in a random order, with a blank and pooled sample analyzed after every four or less drinking water samples. Data was acquired on a Sciex ExionLC ultrahigh performance liquid chromatography system coupled to a Sciex 7600 ZenoTOF high-resolution mass spectrometer. The SPE extracts were separated using RP chromatography using an Acquity UPLC CSH C18 Column (2.1 × 100 mm, 1.7 μm, Waters, Milford, MA, USA) and adapted chromatography conditions from Albergamo et al.^27^ The N_2_ evap extracts were paired with HILIC chromatography using a Zorbax HILIC Plus Column (2.1 × 150 mm, 1.8 μm, Agilent, Santa Clara, CA, USA). Injection volumes for both methods were 10 μL. Data were acquired in both positive and negative ionization modes using data dependent acquisition (DDA) with an electrospray ionization (ESI) source. Detailed chromatography gradients and parameters, quality control measures, and instrumental and analytical performance evaluation are described in the SI and Tables S2 and S3.

### 2.4 Suspect and Nontarget Screening

Initial SS and NTS workflows utilized SciexOS and MS-DIAL with in-house and open-source spectral libraries (MassBank, GNPS), followed by peak alignment, curation, and blank subtraction using a 3× blank intensity cutoff. Full procedural details are given in the SI.

All spectral features matching a spectral library entry (referred to as annotated compounds hereafter) and unidentified compounds without a spectral library match (referred to as spectral features hereafter) were assigned preliminary confidence interval scores in accordance with the Schymanski scale^28^ with minor adjustments. Level 1 confidence includes compounds that match analytical standards based on a matching parent ion mass-to-charge ratio (*m*/*z*), fragmentation spectra, and retention time (±60 s). Level 2a and Level 2b were combined broadly into confidence Level 2 and refer to compounds with matching parent ion *m*/*z*, fragmentation spectra, and retention times that agree with predicted retention time thresholds. Level 3 confidence refers to a compound with a parent ion *m*/*z* and fragmentation spectra that triggered a spectral library match, though measured or predicted retention times were outside the accepted tolerance or were not possible to evaluate. Confidence Level 4 refers to compounds with molecular formulae only, and Level 5 refers to a parent ion *m*/*z* and retention time only.

Confidence Level 1, 2, and 3 annotations were investigated on the United States Environmental Protection Agency’s (US EPA) CompTox Chemicals Dashboard (CCD)^29^ to retrieve chemical identifiers, with the Pubchem database (PubChem)^30^ searched manually when necessary. For 50 of the most abundant ions from the nontarget data set, molecular formulae or tentative structures were generated using SIRIUS and MetFrag, yielding Level 3 annotations.

### 2.5 Data Refinement and Prioritization

#### 2.5.1 Hazardous Chemicals Screening

Hazard screening and data filtering were achieved using the US EPA’s Cheminformatics Hazard Comparison Dashboard (HCD)^31^ combined with an in-house python script (https://github.com/brightpe/Data-Filtering-for-TDW-Project.git) for annotated compounds (Levels 1, 2, and 3). Human health toxicity scores across multiple end points were gathered for 23 annotated compounds, and numerical values were assigned to the chemical’s designated toxicity rank following a previously described approach.^32^ A ‘Total Human Toxicity Score’ was then calculated for each chemical by summing scores across all designated human health end points. More information on how scores are assigned in the HCD has been described previously^33^ and is discussed briefly in the SI with study-relevant examples. Full hazard profiles are included in Table S4. Hazard scores were integrated with abundance profiles, and data were further reduced using theoretical mass error, abundance, and reference spectra criteria outlined in the SI.

#### 2.5.2 Retention Time Prediction

Retention time prediction was performed using the open access python RETIP package to reduce false positive annotations (https://www.retip.app/).^34^ Retention time prediction models were trained using molecular descriptors generated by RDKit for a total of 337 and 289 reference compounds for RP and HILIC methods, respectively, with retention times collected using identical LC–HRMS parameters as the original drinking water analyses. Details on data set handling and final application are described in the SI, with model performance parameters and the testing, training, and external validation data sets included in Table S5. The final Level 1 and 2 annotated data set including data from all samples, pooled sample replicates, and blank replicates is presented in Table S6, while the nontarget data set is presented in Table S7.

#### 2.5.3 Contaminant Confirmation and Semiquantification

Annotated compounds were prioritized based on the Total Toxicity Score, relative abundance, availability of analytical standards, and existing zebrafish toxicity data. Candidate drinking water contaminants were considered verified if the retention time, parent ion *m*/*z*, and fragmentation spectra data profiles matched reference standards. A total of 13 chemical contaminants were confirmed at the Level 1 confidence interval (Table S8), and calibration curves were generated for 12 contaminants to semiquantify extract concentrations. Whole water concentrations were then estimated by normalization to method specific preconcentration factors, followed by blank subtraction. Details including limits of detection are provided in the SI and Table S9a,b.

### 2.6 Zebrafish Morphology and Behavioral Screening

Zebrafish husbandry, chemical exposures, morphological and behavioral assays, and benchmark concentration modeling were conducted using established protocols.^35–38^ Briefly, zebrafish were reared and cared for according to protocols reviewed and approved by the Institutional Animal Care and Use Committee at Oregon State University (IACUC-2024–0510). Wild type (Tropical 5D) zebrafish (*Danio rerio*) embryos were dechorionated and exposed to individual chemicals and sample representative mixtures from 6 h post fertilization (hpf) through 120 hpf. Embryos were assayed for morphological and behavioral responses at 24 hpf, and larvae were assayed at 120 hpf. Benchmark concentrations (BMC_20_) corresponding to a 20% increase in response over control fish were derived using log–logistic concentration–response modeling.^39^ Detailed information on zebrafish husbandry, exposure design and protocol, end point examples and scoring, statistical modeling, and mixture preparation are provided in the SI.

### 2.7 Statistical Analysis

Statistical analysis using principal component analysis (PCA) and hierarchical clustering analysis (HCA) were utilized to address the major compositional differences in the annotated data. The main objectives of these analyses were to identify qualitative patterns specifically with regards to the relationship between the annotated compound abundance profiles and public water system source water types and applied treatment protocols. All analyses were performed within python using the publicly available SciPy and scikit-learn packages. Detailed information on statistical analysis procedures and interpretation of results can be found in the SI.

## 3 Results and Discussion

### 3.1 Chemical Prioritization Confirms Diverse Drinking Water Contaminants

Following data set combination and duplicate removal, just over 3,100 unique spectral features were cumulatively detected (Figures S2b, S3). Despite the 40-fold difference in preconcentration between SPE and N_2_ evap extraction methods, total feature counts were comparable between methods (Figure 1a,b). Initial SS resulted in 245 spectral database matches, which were reduced to 52 Level 2 annotations following retention time prediction and mass error and abundance filtering. Annotated contaminants predominantly were classified as natural products or unclassified use chemicals, with these categories constituting the majority of cumulative spectral feature intensity across drinking water samples (Figure 1c,d). A smaller number of annotated contaminants were assigned to the food additive, personal/household, pesticide, plasticizer, industrial, and transformation product classifications categories. Modeled octanol–water partition coefficient (XLogP) values from PubChem ranged from approximately six to negative six, indicating that combination of the two extraction methods expanded the physicochemical range of annotated contaminants captured in the analysis (Figure S4). Additionally, differences in source water type and applied treatment protocols are captured in PCA sample ordination (discussion of PCA results in SI and Figures S5–S9).

**Figure 1:**
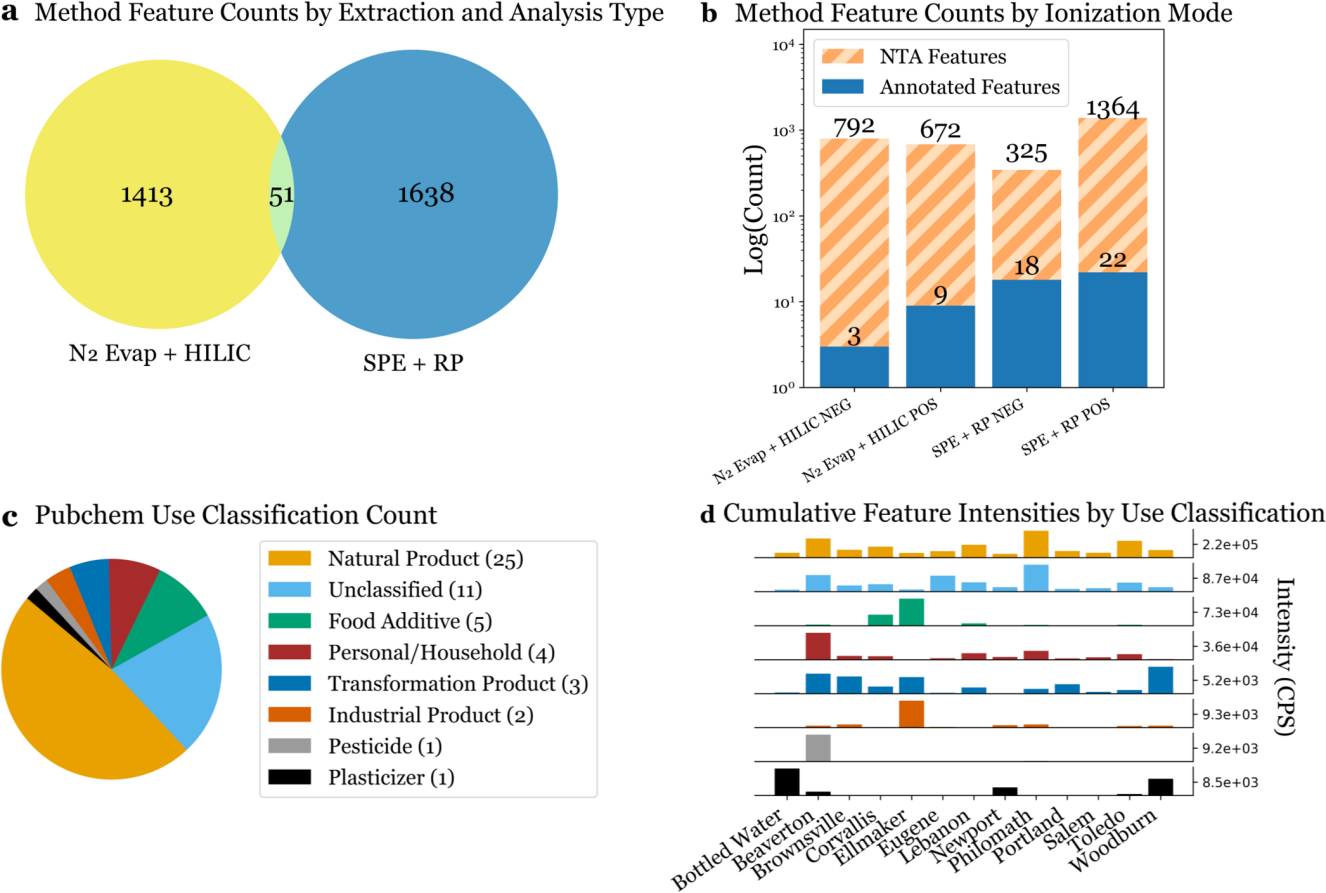
Mass spectral feature overview by extraction method (a) and ionization mode (b). Chemical use classifications were obtained from the PubChem database for all chemical annotations and were used to group data based on the relative contribution of each classification to the total (c), and the cumulative feature intensities of each classification per sampling location (d).

Across the 23 available hazard profiles from the HCD (Figure S10), the most information was available for the route of exposure, genotoxicity, endocrine disruption, and developmental effect end points, with most available data derived from qualitative structure–activity relationship (*QSAR*) modeling rather than *in vivo* toxicity screening or authoritative tier studies. Chemicals with the highest Total Human Toxicity Scores including propiconazole (PCZ), benzyl butyl phthalate (BBP), ethylenediaminetetraacetic acid (EDTA), and saccharin (SAC) were notable exceptions, with most information derived from authoritative and screening tier sources. Chemicals with lower Total Human Toxicity Scores had higher proportions of modeling-based scores or scoreless end points. Contaminants were prioritized for confirmation based on the magnitude of the Total Human Toxicity Score, gaps in toxicity information identified by the HCD profiles, and the frequency of detection. Reference standards were purchased for 30 candidate chemicals spanning confidence Levels 1–3, from which 13 were confirmed by reference standard comparison (Table S8, Figures S11–S23). Relatively large differences between sample and reference standard retention time was noted for the compounds 18β-glycyrrhetinic acid (18β-GA) and atractylenolide III (ATR III), which was partially attributed to matrix effects of SPE extracts in the chromatography system (Figures S24–S27). The potential isomeric interference of 18α-glycyrrhetinic acid (18α-GA) was also considered, with fragmentation spectra indicating 18β-GA was the predominant isomer identified (Figure S11). Semiquantification was conducted for 12 contaminants while EDTA was excluded from quantification due to metal chelating behavior in the LC-HRMS system.

### 3.2 Environmental Context of Well-Known Contaminants

Among the 13 chemical contaminants confirmed at the Level 1 confidence interval through the described workflow, well-studied contaminants including vehicle-related synthetic chemicals, artificial sweeteners, pesticides, and phthalates were observed. Full semiquantified concentration profiles of all confirmed contaminants are provided in Table S9a. Despite the challenges associated with accurate depiction of contaminant concentration profiles through semiquantitative methods (in addition to the recently described effects of household-level variability^20^), broad trends in contaminant presence provide contextual evidence to the types of contamination influencing certain drinking water samples. The best examples from this study are the Ellmaker and Beaverton water samples.

The Ellmaker water sample exhibited the highest estimated water concentration of benzothiazole-2-sulfonic acid (BTSA), and the only confident detections of cyclamate (cyclamic acid, CYC) and saccharin (SAC). The Ellmaker water source is a transient noncommunity water system on Oregon State Highway 20 with a parking lot, bathroom, septic system, and groundwater well located onsite, and represents the least treated water source sampled in this study with routine chlorination the only applied treatment (Oregon State Parks, personal communication, April 4, 2025). Benzothiazoles are recognized environmental tracers of road-runoff,^40,41^ and together with the artificial sweeteners CYC and SAC, serve as tracers of wastewater influence in hydrologic systems.^42–44^ Specifically, BTSA is a transformation product of the rubber vulcanization agent 2-mercaptobenzothiazole (2-MBT) used in tire manufacturing, with both BTSA and 2-MBT demonstrated to leach from tire particles in aqueous suspension.^45^ Detections of BTSA have been documented in estuary water, road and stormwater runoff, wastewater, and drinking water.^41,46–48^ Similarly, artificial sweeteners CYC, SAC, sucralose (SUC; identified at Level 2 confidence in this study), and acesulfame (ACE; evidence of presence in this study) have reported detections in wastewater, surface waters, and drinking waters, in part due to minimal biotransformation that occurs during human metabolism and the relatively high water solubility of these compounds.^42^ Estimated water concentrations of CYC and SAC in the Ellmaker water sample (0.7 and 37.0 ng/L, respectively) are well below concentrations reported for surface waters (<130 ng/L CYC, < 380 ng/L SAC^42^), shallow groundwater aquifers with known wastewater influence (<1,200 ng/L CYC, < 10,000 ng/L SAC^43,49^ ), and WWTP influents and effluents (can both be greater than 10,000 ng/L^42,44^ ). However, BTSA was detected at an estimated water concentration of 1,330 ng/L in the Ellmaker water sample, which comparatively surpasses that seen in estuarine waters (∼800 ng/L^47^) but remains below the reported range in wastewater (≤1,700 ng/L^40^). Combined detection of BTSA, CYC, SAC, and SUC suggests the Ellmaker water source is influenced by ongoing road-runoff and wastewater related inputs, as detection of CYC in water samples in the United States is unusual (CYC was banned for use as an artificial sweetener in the U.S in the 1970s^50^), and storage studies have demonstrated substantial decline of CYC concentrations within 3 weeks and complete loss of both CYC and SAC within a year in refrigerated storage.^49^ Furthermore, PCA and HCA results suggest this water sample is compositionally unique from other waters sampled in this study, characterized by a relative lack of triterpenoid natural compounds and overall lower cumulative annotated feature intensity, potentially resulting from highly local but limited sources of contaminants paired with minimal treatment (Figures S5 and S6).

The herbicide transformation product metolachlor ethanesulfonic acid (MESA) and the fungicide PCZ were both detected at their highest estimated concentrations in the Beaverton water sample. Additionally, the Beaverton sample showed the highest estimated concentrations of natural products N-acetylglutamic acid (NAG), 18β-GA, and several other Level 2 confidence triterpenoid natural products contributing to its distinct composition and PCA ordination. The Beaverton public water system distributes water to around 80,000 people and primarily operates on pretreated Tualatin River derived drinking water received from the Joint Water Commission (JWC), supplementing from local wells when necessary.^51^ The Tualatin River has faced historical water quality issues and was the first water body in the U.S. to be regulated under the Federal Clean Water Act, with the watershed influenced largely by urban wastewater, agricultural, and forestry related inputs.^52^ The herbicide metolachlor is used to eradicate broadleaf weed species including soybean, corn, peanut, cotton, safflower, potato, and peanut crops, with its metabolic breakdown product MESA a persistent glutathione conjugate of this chemical.^53–55^ Similarly, the curative fungicide PCZ is used agriculturally for crops including soybeans and corn with additional nonagricultural uses such as for golf course and lawn care.^54–57^ Both MESA and PCZ can remain in soils for hundreds of days to years and are exceedingly common in hydrologic systems. For example, an Iowa based series of studies detected MESA in 99% of surveyed stream samples and MESA degradation products in nearly 75% of groundwater samples, while PCZ has documented occurrences in streams, groundwater, lakes, and drinking water.^54,56,58,59^ In this study, MESA was detected in five samples across the study spanning water systems operating from surface water, groundwater, and a mix. In contrast, PCZ was detected only in the Beaverton water sample. In addition to high cumulative intensity of triterpenoid natural products observed in the Beaverton sample, the co-occurrence of MESA and PCZ suggests this water sample is notably influenced by agricultural-related inputs.

Three other well-known synthetic chemicals were detected in this study including benzyl butyl phthalate (BBP), the BBP hydrolysis product monobenzyl phthalate (MBzP), and ethylenediaminetetraacetic acid (EDTA). Phthalates are known environmental contaminants due to use as plasticizers and their predisposition to leach from materials after synthesis,^60–62^ and EDTA has varied uses leading to contamination of surface waters and drinking waters, with pulp and paper manufacturing described as a major source to the environment.^63,64^ A single detection of BBP was observed in the bottled water sample, while MBzP was detected in four total samples. In this study, the bottled water sample is the only sample sourced from a polyethylene terephthalate (PET) bottle and represents the most thoroughly treated sample including reverse-osmosis and ultraviolet sterilization processes, likely contributing to the unique detection of BBP and compositionally unique character of this water sample captured by PCA. Furthermore, detections of phthalates in PET bottled water have been reported previously at concentrations ranging from tens of ng/L to more than 50 μg/L,^61,65,66^ indicating a leaching-based source is plausible. The detection of MBzP in multiple water samples may indicate environmental input and transformation of BBP in source waters, as BBP is expected to have a short half-life in aerobic aquatic systems where biodegradation occurs.^67^ While BBP, MBzP, and EDTA have disparate uses, all are synthetic chemicals indicative of industrial influence in drinking water sources.

### 3.3 Novel Drinking Water Contaminants

In addition to the well-known contaminants described above, a suite of five confirmed chemicals detected in this study are novel drinking water contaminants. To the best of our knowledge, this study reports detections of NAG, taurochenodeoxycholic acid (TCDCA), 18β-GA, ATR III, and quinoline-4-carboxylic acid (QCA) in drinking water for the first time. Apart from the synthetic chemical QCA, these contaminants are derived from natural products including an acetylated amino acid (NAG), a bile acid conjugate (TCDCA), and plant-derived compounds associated with licorice root (*Glycyrrhiza glabra*) and Cang Zhu (*Atractylodes lancea*) extracts (18β-GA and ATR III, respectively). Routes of drinking water contamination are not established for these five compounds. However, QCA is synthetically useful in medicinal chemistry,^68^ with its structure incorporated in current market pharmaceuticals such as ciprofloxacin and chloroquine.^69,70^ Further, QCA is listed as a chemical of emerging concern by the European Human Biomonitoring Initiative and is included in the Blood Exposome Database, highlighting its relevance to environmental occurrence and human exposure.^71,72^ Otherwise, TCDCA, 18β-GA, and ATR III all occur in traditional medicine, particularly in Asian cultures, and have been studied for a suite of therapeutic properties.^73–77^ Cosmetic uses of NAG and 18β-GA are approved by the Cosmetic Ingredient Review Expert Panel,^78,79^ and both NAG and 18β-GA occur naturally in foodstuffs such as coffee and licorice, respectively, with reported concentrations of NAG reaching the μg/g range,^80^ potentially contextualizing the estimated concentration range of NAG in drinking water observed here (<8.3 μg/L). Oral administration of glycyrrhizin (the hydrolysis precursor of 18-GA) and ATR III resulted in detectable amounts of 18β-GA and ATR III in the urine of humans and rats, respectively, and NAG and 18β-GA have been detected directly in human urine previously.^81–83^ Therefore, drinking water contamination through a surface water related infiltration pathway is plausible, as all five chemicals occurred at their relative maximum estimated concentrations in drinking water derived from surface water sources.

### 3.4 Zebrafish Screening Indicates Potent Toxicity for Select Contaminants

Eight confirmed drinking water contaminants were individually screened for toxicity using the zebrafish embryo and larvae assay. The novel drinking water contaminants NAG, TCDCA, 18β-GA, ATR III, and QCA were prioritized for screening given toxicity information available for these chemicals overall is limited, and the available HCD profiles are based entirely on predictive modeling and have nonscored end points. Known drinking water contaminants BTSA, MBzP, and MESA were prioritized based on the lack of individual screening in the zebrafish model, though BTSA and MESA were previously screened with zebrafish as part of chemical mixtures.^41,84^ Zebrafish toxicity screening data for BBP, PCZ, and SAC are presented for reference but were not rescreened for this study.^85–87^

Toxicity screening for each chemical included morphological and behavioral toxicity evaluation over biologically active concentration ranges, followed by sample representative mixtures screening. Chemical mixtures were prepared by adjusting contaminant concentrations until the most concentrated Level 1 contaminant in each sample reached a concentration of 10 mM, while preserving molar ratios to other contaminants (Figure 2, Table S10). Morphological BMC_20_ values and behavioral outcomes are summarized in Figure 3, with full output and end point abbreviations provided in Table S11a–c.

**Figure 2:**
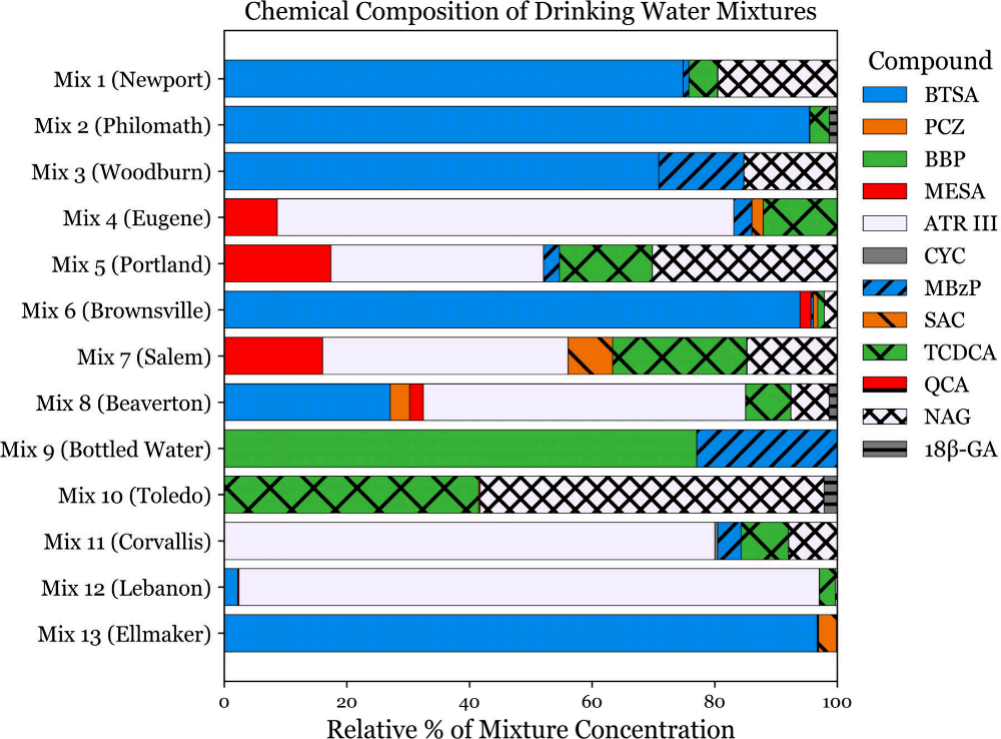
**C**hemical mixture compositions used for zebrafish toxicity screening to evaluate mixture-based toxicity of drinking water contaminants. The relative percent (%) contribution of individual contaminants to each mixture (total = 100%) is shown, with mixture compositions tabulated in Table S10. Chemical abbreviations: Benzothiazole-2-sulfonic acid (BTSA), Propiconazole (PCZ), Benzyl butyl phthalate (BBP), Metolachlor ESA (MESA), Atractylenolide III (ATRIII), Cyclamate (CYC), Monobenzyl phthalate (MBzP), Saccharin (SAC), Taurochenodeoxycholic acid (TCDCA), Quinoline-4-carboxylic acid (QCA), N-Acetylglutamic acid (NAG), and 18β-Glycyrrhetinic acid (18β-GA).

**Figure 3:**
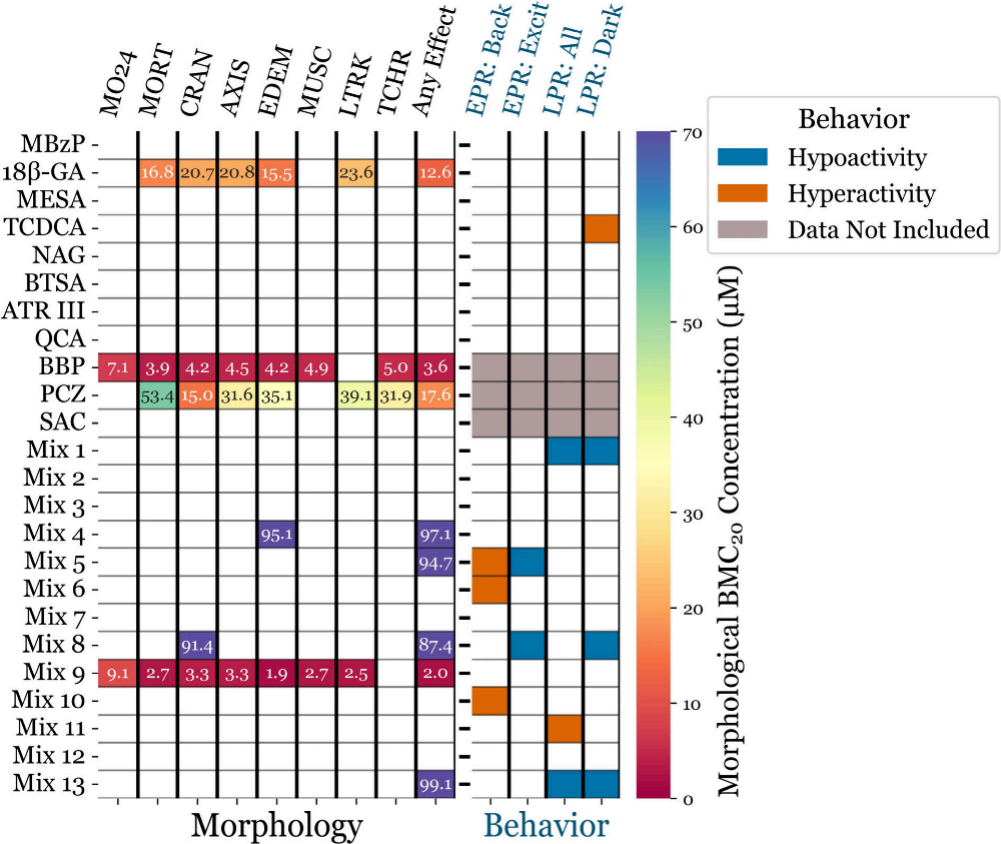
Zebrafish responses across morphological and behavioral end points following neat chemical and chemical mixture exposures. Morphological BMC_20_ values are shown for end points with observed incidence and are color-coded according to the heatmap scale. Behavioral outcomes are displayed categorically, with colors indicating hypoactivity or hyperactivity. End points shown include those with at least one observed incidence; additional tested end points without responses and behavioral BMC_20_ values are provided in Tables S11a and S11b, and end point abbreviations are defined in Table S11c.

#### 3.4.1 Developmental Toxicity and Mortality

Among the screened chemicals, 18β-GA produced adverse morphological effects across six end points in the 16–24 μM BMC_20_ range, including mortality. No morphological effects were observed for the other seven tested chemicals, though previously reported morphological abnormalities are noted for BBP and PCZ, where BBP is the most potent toxicant in the study with BMC_20_ values in the 4–7 μM range.^85^

Exposure to 18β-GA resulted in pronounced toxicity in developing zebrafish. In humans, licorice toxicity is mediated primarily by 18β-GA and glycyrrhizin, manifesting as apparent mineralocorticoid excess including symptoms like hypertension, hypokalemia, and heart failure.^81,88,89^ Inhibition of 11-β-hydroxysteroid dehydrogenase type 2 (11-β-HSD2) and 5 β-reductase result in aldosterone and cortisol accumulation and hyperstimulation of the mineralocorticoid receptor (MR).^90^ In zebrafish, a single glucocorticoid receptor (GR) and MR have been identified, where the GR appears to perform fluid and ion homeostasis roles typical of the MR in mammals, and aldosterone is absent while cortisol serves as the primary GR ligand.^91,92^ The observed edema, craniofacial malformations, and body axis abnormalities in this study following 18β-GA exposure may indicate parallel GR and MR overstimulation via cortisol accumulation, consistent with a prior observation where 1.0 mg/L cortisol exposure lead to craniofacial malformations attributed to changes in GR-mediated matrix metalloproteinases.^93^ Morphological BMC_20_ values beginning at 12.6 μM (5.9 mg/L) 18β-GA exposure fall within the same order of magnitude as the reported cortisol-mediated developmental effects, though verifying consequent activity of the GR and MR would require further mechanistic investigation. The lowest BMC_20_ exposure concentration determined for 18β-GA exceeds the maximum semiquantified drinking water concentrations detected in this study (4.5 ng/L) by roughly six orders of magnitude, and the full extent of human exposure cannot be assessed from occurrence data alone. However, the potent flavor and historically recognized medicinal properties of licorice have led to its incorporation into foods, beverages, and medications, though cases of toxicity and hospitalizations from high consumption are documented worldwide.^73,88,90^ Although drinking water consumption likely represents a minor exposure route given 18β-GA’s low water solubility (20 mg/L, Table S8), its incremental contribution to human exposure warrants further consideration for water monitoring.

Toxicity in zebrafish following exposure to the BBP and PCZ have been reported previously,^85,87,94–96^ with BBP recently designated by the US EPA as posing unreasonable risk to human health under certain conditions of use.^97^ The BMC_20_ values under BBP exposure in Rivera et al. start at 3.6 μM (1.1 mg/L),^85^ considerably exceeding the semiquantified concentration of BBP in the bottled water sample (2.75 ng/L). Similarly, the range of BMC_20_ values observed under PCZ exposure span 15.0 to 53.4 μM (5.3 to 18.3 mg/L),^87^ considerably exceeding the maximum concentration of PCZ semiquantified in drinking water (8.3 ng/L). Nevertheless, the presence of BBP and PCZ in drinking water remains notable given their established toxicity profiles. Otherwise, no significant morphological or behavioral toxicity was observed for BTSA, MESA, and five other chemicals screened in this study, contrasting previous observations of aquatic toxicity following exposure to the BTSA precursor 2-mercaptobenzothiazole^40,41,46^ and morphological toxicity in zebrafish embryo and larvae following MESA exposure.^84^ Here, no significant toxicity was observed following exposures up to 100 μM of either BTSA or MESA.

In this study, MBzP, a primary phase I metabolite of BBP, was screened for toxicity due to a low volume of toxicity data and information available compared to BBP. No significant morphological or behavioral toxicity was observed for neat MBzP exposures up to 100 μM (25.6 mg/L). However, exposure to a mixture of MBzP and BBP in a 3:10 proportion (representative of the bottled water sample, Mix 9) presented more potent toxicity than exposure to BBP alone in all end points except mortality at 24 hpf, with malformations in the trunk and caudal fin unique to this mixture. Given BBP is largely metabolized to MBzP in humans,^98^ coexposure may result in additive or mildly synergistic toxicity. Moreover, mortality at 120 hpf, craniofacial and body axis abnormalities were between 20 and 40% lower than under BBP exposure alone. These findings suggest a potential role of MBzP in mixtures toxicity, consistent with a previous observation of trunk and caudal fin abnormalities following exposure to a phthalate mixture including MBzP, but lacking BBP.^99^

Morphological effects were observed in four additional contaminant mixtures representing Eugene (Mix 4), Portland (Mix 5), Beaverton (Mix 8), and Ellmaker (Mix 13). Observed BMC_20_ values ranged from 87 to 99 μM for the cumulative Any Effect end point, with additional edema and craniofacial abnormality end points observed in the Eugene and Beaverton mixture exposures, respectively. Morphological effects were not strongly correlated with cumulative total contaminant concentration, suggesting mixture composition is a more important driver of bioactivity. The relatively high proportion of PCZ in the Beaverton mixture potentially explains the observed craniofacial effects, though the Eugene, Portland, and Beaverton mixtures were dominated by ATR III with varying amounts of MESA, TCDCA, and QCA, where none of these chemicals produced toxicity when tested individually. Overall, these results highlight the bioactive complexity of chemical mixtures and the limitations imposed by the lack of established LC_50_ or BMC_20_ values.

### 3.5 Contaminant Mixture Exposures Result in Behavioral Abnormalities

Abnormal zebrafish behavior was observed following exposure to TCDCA and seven sample representative mixtures in the larval photomotor response (LPR) and embryonic photomotor response (EPR) assays (Figure 3). The LPR and EPR assays provide sensitive and sublethal measures of bioactivity using light pulses to evoke movement.^100^ A key difference between LPR and EPR assays is developmental stage: larvae have undergone organogenesis and development of xenobiotic metabolism capability and respond to light pulses through visual stimulation, whereas embryos have not developed eyes or xenobiotic metabolizing capability and respond to light pulses via photoreceptor activity.^35,37^ Exposure to TCDCA resulted in hyperactivity during the dark phase of the LPR assay, with a calculated BMC_20_ of 28.9 μM (14.5 mg/L), without corresponding abnormal behavior in the EPR assay. Abnormal behavior at 120 hpf can indicate impairment of larval biological systems during organogenesis,^101^ consistent with the recognition of TCDCA as a bioactive substance.^75,76^ Behavioral abnormalities were also observed in both LPR and EPR assays following exposure to seven sample representative mixtures. In the LPR assay, hypoactivity was observed under exposure to the Newport (Mix 1), Beaverton, and Ellmaker mixtures (3–40 μM BMC_20_ range), while hyperactivity was observed following exposure to the Corvallis mixture (Mix 10, BMC_20_ of 41.4 μM). In the EPR assay, hypoactivity was observed in the Portland and Beaverton mixture exposures (BMC_20_ 10–50 μM) while hyperactivity was also observed in the Portland mixture exposure, as well as in the Brownsville (Mix 6) and Toledo (Mix 10) exposures (BMC_20_ values 68–75 μM). Notably, BTSA, TCDCA, and NAG were common components among the bioactive mixtures but did not outright predict bioactivity, as compositionally similar mixtures such as Newport and Philomath (Mix 2) were bioactive and nonbioactive in the LPR assay, respectively, with similar observations in the EPR assay (e.g., Portland and Salem (Mix 7)). Furthermore, TCDCA was the only chemical to induce abnormal behavior individually but did not induce abnormal behavior in some mixtures (e.g., Salem and Eugene). Again, these results demonstrate the complexity of mixture-driven bioactivity. Therefore, the importance of accounting for mixture effects in drinking water exposure assessments is highlighted.

### 3.6 Analytical Method Evaluation and Proposed Adjustments

Analytical constraints in SS results highlight methodological adjustments that could improve the annotation efficiency in larger analyses. While mass error, abundance, and retention time prediction thresholds were imposed to conservatively eliminate false positives, just two percent of the cumulative 3,200 mass spectral features detected were confidently annotated at Level 1 and 2 confidence. Of the 52 annotated compounds, 28 produced a single diagnostic fragment peak (defined here as a peak greater than or equal to five percent of the parent ion peak, bringing it above background), with no observed relationship of hydrophobicity (XlogP value), mass, or ionization mode to fragmentation peak quantity observed. Data acquisition at multiple collision energies could likely increase accurate spectral library match rates, especially given that the libraries employed in this study contained hundreds of thousands and tens of thousands of spectral entries for positive and negative ionization modes, respectively. In addition to expanded fragmentation data, annotation efficiency could be improved by matching library compositions to both ionization mode and the general physicochemical range isolated by extraction and analysis methods, as the RP and HILIC protocols employed here covered unique ranges of XlogP values (Figure S4). Finally, matching mobile phase composition to both ionization mode and LC separation method could improve holistic coverage of the chemical space present in samples. In this study, mobile phases were optimized for LC separation methods but kept constant between ionization modes to streamline data set combination, potentially contributing to the comparatively low spectral feature counts in negative mode analyses, particularly in the RP analysis (325 in negative mode versus 1,364 in positive mode RP analysis).

### 3.7 Limitations

As a pilot study, several limitations must be acknowledged when considering the significance and novelty of the findings. The *in vivo* toxicity data presented here are intended to inform hazard-based risk assessments of specific contaminants detected in drinking water, and not the drinking water itself. Simplified mixtures and neat chemical exposures do not capture the full chemical complexity of drinking water, and reported BMC_20_ values here apply to the chemicals and mixtures tested, not the drinking waters sampled. Application of effect-directed analysis to whole and concentrated drinking waters could be a beneficial alternative approach to assessing whole water-based toxicity, with bioactivity driving identification of the most toxic substances in drinking waters. Further, human exposure to the contaminants identified in this study remains ambiguous; coordinated evaluation of drinking water, human excretion pathways, and wastewater are needed to better understand exposure and associated bioactivity. Species-specific differences must also be noted and may limit the direct extrapolation of observed toxicity to human health outcomes. In the context of this study, differences in human biology, particularly with respect to MR and GR processes, may reduce the potency of 18β-GA toxicity in humans. Finally, the limited sample size constrains conclusions regarding contaminant occurrence and sources, and the semiquantitative concentration estimates overlook explicit measures of extraction efficiency, therefore potentially underestimating true concentrations in samples in an inconsistent and unknown manner. Future studies could mitigate these challenges by employing a broad physicochemical range of extraction standards (or via a matrix spike as performed elsewhere)^48^ to account for loss during sample collection and processing, and by expanding the sampling range and replication.

### 3.8 Significance

This pilot study demonstrates a data-driven workflow that integrates suspect and nontarget screening with publicly available hazard data to guide *in vivo* toxicity testing of contaminants detected directly in drinking water. The use of complementary extraction and chromatographic methods expanded detection across a broader range of organic contaminants, enabling the identification of both known and previously unrecognized contaminants. With the exception of 18β-GA, the most toxic chemicals detected in this study are well-characterized environmental contaminants with established toxicological profiles, whereas the lesser-known or novel contaminants lacked toxicological information, relying mostly on model-based hazard estimates. Notably, this approach generated the first *in vivo* BMC_20_ values for 18β-GA, which produced adverse morphological toxicity in zebrafish larvae comparable to better known toxicants such as the fungicide propiconazole. Additive to mildly synergistic toxicity following coexposure to BBP and MBzP in zebrafish was also novelly observed here. Collectively, the suspect and nontarget screening and *in vivo* toxicity findings presented here underscore the value of integrated analytical and toxicological approaches for advancing evaluations of drinking water quality.

## Supplementary Material







## Data Availability

Raw mass spectral data files for the tap drinking water analyses can be found in the Oregon State University Scholars Archive at 10.7267/ww72bm59t.

## Notes

Sample extraction protocols, chromatography, and mass spectrometer conditions; suspect screening protocol including use of the US EPA Hazard Comparison Dashboard, retention time prediction, final quality control; semiquantification description; zebrafish husbandry and chemical exposure information; statistical analysis including procedure and interpretation; sampling map; sample processing schematic; suspect and nontarget data overview and retention time comparison; 13 chemical fragmentation spectra comparison plots (PDF)
Public water system details; list and performance metrics of utilized deuterated internal standards; mass spectrometry parameters; Hazard Comparison Dashboard output profiles; retention time prediction data sets and performance metrics; final annotated data set; semiquantification information; contaminant mixture information; zebrafish BMC_20_ values and confidence intervals (XLSX)
Nontarget analysis study reporting tool (XLSX)
