# Supplementary material for: Integrated Chemical and Toxicity Screening of Tap Drinking Water across Western Oregon Using Suspect and Nontarget Screening
Source: Environ Sci Technol. 2026 Jun 17;60(25):17706–17. doi: 10.1021/acs.est.6c01661 (PMC13325860; doi:10.1021/acs.est.6c01661)
Supplement: Supplementary file 1 [file es6c01661_si_001.pdf]

Integrated Chemical and Toxicity Screening of Tap Drinking Water across Western Oregon using Suspect and Non-Target Screening

Peter W. Bright[Ø], *Miranda E. Jackson, Chloe L. Fender, Kenneth Lee, Caoilinn Haggerty, Jason Schindler, Lisa Truong, Robyn L. Tanguay, Manuel Garcia-Jaramillo**

Department of Environmental and Molecular Toxicology, Oregon State University, Corvallis, OR 97331.

[Ø] Lead Author

*Corresponding Author

Email Address: *manuel.g.jaramillo@oregonstate.edu*

Number of Figures: 27

Total number of pages: 43

METHODS **2**

Sample Extraction Details. **2**

Solid Phase Extraction *2*

Nitrogen Gas Evaporation. *3*

UPLC-HRMS Analysis: Chromatography Details. **4**

Reverse Phase Chromatography. *4*

Hydrophilic Interaction Chromatography. *4*

UPLC-HRMS Analysis: Mass Spectrometer Details. **5**

Instrumental and Analytical Performance. **5**

Suspect and Non-target Screening. **5**

Hazard Comparison Dashboard Information **6**

Mass Error, Abundance, and Reference Spectrum Quality Control. **7**

Retention Time Prediction. **8**

Semi-quantification, Limit of Detection, Limit of Quantification. **9**

Zebrafish Morphology and Behavioral Screening. **10**

Zebrafish Husbandry. *10*

Chemicals Screening. *11*

Sample Representative Chemical Mixtures. 13
Statistical Analysis. 14
Principal Component Analysis. 14
Hierarchical Clustering Analysis. 14
RESULTS and DISCUSSION 15
Statistical Analysis: Patterns of Contaminants in Drinking Waters. 15
Patterns in PCA and HCA. 15
SUPPLEMENTAL FIGURES. 19
REFERENCES 41

METHODS

Sample Extraction Details.

Solid Phase Extraction. Cartridge solid phase extraction (SPE) was performed using the automated PromoChrom system (SPE-003 MOD-00P, PromoChrom Technologies LTD) equipped with 1000 mg 20 mL HLB SPE cartridges (Waters Oasis, 80 Å pore size, 60 µm particle size, Part No: 186000117). The PromoChrom system was pre-conditioned with 20% LCMS grade methanol (MeOH) in ultrapure water according to the manufacturer's recommendation prior to sample extractions. Water samples were transferred to precleaned 1 L high density polyethylene (HDPE) bottles compatible with the PromoChrom system and allowed to warm to ambient temperature prior to extraction. The SPE cartridges were first conditioned with 10 mL MeOH, followed by 10 mL ultrapure H_2O acidified to pH 2-3 using formic acid (H_2O-FA), after which 1000 mL of sample was loaded onto the columns at a flow rate of 10 mL/min. Following sample loading, sample bottles were rinsed with 8.0 mL LCMS grade water, and the bottle 'rinsate' was then passed through SPE columns at 5 mL/min. Columns were then dried for 15 minutes using a gentle stream of air. The walls of the bottles were rinsed again with 15 mL of MeOH, and samples were then

eluted from SPE columns using the 15 mL of MeOH bottle rinsate at a flow rate of ~10 mL/min. Eluates were collected into 15 mL polypropylene (PP) centrifuge tubes, and nitrogen gas (N_2) was passed through the columns to ensure all elution solvent was collected. Extracts were concentrated to a target 800 µL by evaporation under a gentle N_2 stream at 30°C using a 48-position multivap nitrogen evaporator (Organomation, #11848). Extracts were then transferred to reduced surface area glass autosampler vials (Cat. No. 9532S-OCV-RS RSA, Microsolv Technology Corporation), and the extract tubes were rinsed with 200 µL MeOH that was subsequently added to the sample. Final sample volumes were adjusted as necessary to achieve 1000 µL with MeOH for a final preconcentration factor of 1000x.

Nitrogen Gas Evaporation. Nitrogen gas evaporation (N_2 evap) was performed by pipetting a 10 mL aliquot of each water sample into a 15 mL glass centrifuge tube (DWK Life Sciences, Catalogue No. 73790-15). The centrifuge tube was placed in an automated evaporation apparatus with a gentle stream of N_2 and a water bath set to 60°C to evaporate samples to a volume of 200 µL. The evaporate was transferred to a 1.7 mL PP centrifuge tube, and ~200 µL of acetonitrile (ACN) was added to adjust the final volume to 400 µL, resulting in a final sample preconcentration factor of 25x in a 50:50 ACN:H_2O composition.

For each extraction method, a pooled sample was prepared by adding 20 µL of each sample to a glass RSA autosampler vial, and a process blank was prepared with ultrapure water using the same extraction process. Extracts from all methods were spun at 14,000 rpm for 20 minutes following extraction, transferred to a new autosampler vial, and were stored at 4°C until analysis. Finally, a 200 µL aliquot was added to a 300 µL glass autosampler vial with 100 µg/L isotopically

labelled internal standards; 6-nitrochrysene-D11, 9,10-anthraquinone-D8, and imidacloprid (4,4,5,5-D4) for positive mode SPE extracts, imidacloprid (4,4,5,5-D4) and salicylic acid-D4 for negative mode SPE extracts, imidacloprid (4,4,5,5-D4) for positive mode N_2 evap extracts, and imidacloprid (4,4,5,5-D4) and 2,4,6-trichlorophenol-D2 for negative mode N_2 evap extracts (See **Tables S2a and S2b** for analytical standards information and performance).

UPLC-HRMS Analysis: Chromatography Details.

Reverse Phase Chromatography. The SPE extracts were separated using reverse phase (RP) chromatography using an Acquity UPLC CSH C18 Column (2.1x100 mm, 1.7 µm, Waters, Milford, MA, USA) with mobile phases of LC-MS grade water (A) and LC-MS grade MeOH (B) both acidified with 0.1% formic acid. Separation of 10 µL sample injections was performed with a gradient elution that started at 10.0% B until 1.00 minute, ramped to 50.0% B by 4.00 minutes, then slowly ramped to 95% B by 17.00 minutes. The gradient remained at 95% B until 25.00 minutes before returning to 10.0% B by 25.10 minutes to equilibrate until the end of the run at 30.00 minutes (chromatographic method adapted from Albergamo et al[1]). The flow rate was a constant 0.300 mL/min and the column temperature was held constant at 50°C throughout the separation.

Hydrophilic Interaction Chromatography. The N_2 evap extracts were paired with hydrophilic interaction chromatography (HILIC) using a Zorbax HILIC Plus Column (2.1x150 mm, 1.8 µm, Agilent, Santa Clara, CA, USA) with mobile phases consisting of 95:5 ultrapure water:ACN (A) and 95:5 ACN:ultrapure water (B), both with 5mM ammonium formate, adjusted to pH 3 with formic acid. Separation of 10 µL sample injections was performed with a gradient elution that started at 95% B from 0.00 to 4.00 minutes, decreased to 90% B by 15.00 minutes, then decreased to 20%

B by 21.00 minutes before returning to 95% B by 22.00 minutes to equilibrate until the end of the run at 30.00 minutes.

UPLC-HRMS Analysis: Mass Spectrometer Details. Data were acquired for all samples in both positive and negative ionization modes using "information dependent acquisition" (IDA; also known as data dependent acquisition, DDA) within Sciex using an electrospray ionization (ESI) source. For both positive and negative acquisition modes a precursor ion TOF MS scan ranged from 50-1,000 Dalton (Da) with a TOF MSMS scan from 40-1,000 Da. Carrier gasses were set to 50 psi, the curtain gas was set at 35 psi, and the source temperature was 500°C. Other instrument parameters are given in **Table S3**.

Instrumental and Analytical Performance. During analysis, the instrumental mass accuracy was auto calibrated every five samples using Sciex mass analyzer calibration solutions. Replicate analytical standard mixes were analyzed at the beginning and end of each sample analysis to constrain differences in instrument performance across the run. Relative standard deviations (RSD) of the chromatographic peak areas across the duplicated standard mix analyses were ±11% or below for all standards in all analyses (**Table S2a**). Solvent blanks and pooled samples were analyzed every four samples to ensure acceptable instrumental performance. The RSD of all internal standards across samples and pooled sample replicates was less than ±25% across analyses, with the exception of 37% RSD for the deuterated-Imidacloprid (4,4,5,5-D4) internal standard in positive mode RP analysis.

Suspect and Non-target Screening. Raw mass spectral data files (.wiff2 files) were converted to Analysis Base File (ABF) format using Reifycs ABF converter (Tokyo, Japan) before being imported

into MS-DIAL, version 4.0.7.[2] Initial suspect screening for each instrumental run was executed in SciexOS using an in-house spectral library consisting of spectra acquired from the IROA Mass Spectrometry Metabolite Library of Standards (MSMLS, IROA Technologies, Ann Arbor, MI), and metabolites and xenobiotics previously detected in-house. Initial annotations made with in-house libraries were then incorporated into the subsequent peak alignment and data curation in MS-DIAL.[2] Additional annotations were made in MS-DIAL using the open-source MS-DIAL metabolomics library including MassBank and GNPS (Global Natural Product Social Molecular Networking) libraries (MSP spectral kit, V. 17). Following suspect screening and spectral matching, blank subtraction and data curation was performed in Microsoft Excel. Blank subtraction was performed by flagging and excluding all features with average sample peak intensities less than three times the greatest feature intensity detected in the blank, extraction blank, or field blank.

Hazard Comparison Dashboard Information. Output from the U.S. Environmental Protect Agency (US EPA) Hazard Comparison Dashboard (HCD) contains risk scores for the individual water contaminants based on a five-point tiered system spanning very high, high, medium, low, and inconclusive risk. These scores are based on bounded assay values for lethal concentration affecting 50% of the test population (LC_{50} values), no observed adverse effects level (NOAEL) values, and other endpoints as described in Vegosen and Martin.[3] Each hazard score is additionally marked with the relevant assay and authority of designation associated with the given risk score, spanning authoritative, screening, and quantitative structure-activity relationship (*QSAR*) designations. Authoritative sources describe toxicity designations that have been established and recognized by professionals and authoritative sources such as government and regulatory agencies. The screening tier describes data that is derived from a less

authoritative source or less comprehensive studies, while the *QSAR* Model or predictive tier refers to data sourced from the US EPA's Toxicity Estimation Software Tool (T.E.S.T.), which uses structure activity relationships to predict toxicity.[3]

The highest total human toxicity score calculated for compounds detected in drinking water screened in this study belonged to the fungicide propiconazole (PCZ). Very high or high toxicity risk scores were assigned in the inhalation route of exposure, carcinogenicity, endocrine disruption, reproductive and developmental effects, eye irritation, and systemic toxicity associated with repeat exposures categories (**Fig S10; Table S4**). Further, toxicity designations are derived from sources in the authoritative tier, such as the Toxval initiative of the US EPA and multiple other screening tier studies.[4] Similarly, toxicity scores designated for benzyl butyl phthalate (BBP), ethylenediaminetetraacetic acid (EDTA), and saccharin (SAC) are derived almost exclusively from authoritative and screening tier studies. For example, the BBP hazard profile has high scores for the endocrine disruption, reproductive toxicity, and developmental toxicity categories from authoritative sources, and very high toxicity scores derived from the ToxVal Database and individual screening studies based on the incidence of a cancer slope factor.[5] In comparison, the EDTA and SAC hazard profiles have less authoritative information available, but notably more than half of the toxicity category scores include screening tier study data. All other compounds had higher proportions of screening based, modelling based, or non-scored endpoints.

Mass Error, Abundance, and Reference Spectrum Quality Control. A theoretical mass error associated with the detected mass-to-charge (*m/z*) ratio was calculated for each annotated compound. Compounds with greater than 20 ppm error were removed from the annotated

dataset. Similarly, low abundance annotations (< 200 cps in the average of pooled samples) and features with parent or fragmentation reference spectra derived from blank or internal standard samples were removed. Where possible, a theoretical mass error was calculated for the non-target dataset, and the same abundance and reference spectrum criteria were used to reduce the non-target dataset.

Retention Time Prediction. To reduce the number of false positive annotations, the open-source retention time prediction software package RETIP was applied to the dataset to adjust confidence scores for the annotated compounds.[6] First, a group of metabolites sourced from the MSMLS including carboxylic acids, amino acids, nucleotides, and enzymes were analyzed using identical UPLC-HRMS instrumentation and parameters utilized for drinking water sample analysis. In total, retention times for 337 compounds were collected across positive and negative ionization modes with RP analysis, and 289 compounds were detected across both polarity modes in HILIC analysis.

Following peak integration and retention time determination, the data were organized, retention times were log-scaled for use in RETIP, and molecular descriptors for each chemical were gathered using the python toolkit RDkit (https://www.retip.app/).[6] Datasets were then split into training and testing datasets in an 80:20 proportion, and retention time models were created for the training datasets using four individual machine learning algorithms; random forest, extreme gradient boosting, automated machine-learning, and H_2O automated machine-learning.[7] Performance metrics including the root-mean-square error (RMSE), mean absolute error (MAE), coefficient of determination (R^2), and the 95% confidence interval in minutes were calculated for each model's training and testing dataset performance. External validation datasets were used to evaluate model predictions for known compounds.

For each chromatographic dataset, the model with the best performance as evaluated by error metrics and retention time prediction accuracy against the external validation dataset was utilized to predict retention times. Compounds with predicted retention times within the retention time error range of Level 1 compound predictions or the modeled 95% confidence interval for the external validation dataset were kept in the Level 2 confidence interval. Compounds that fell outside this range were moved to the mass spectral feature dataset and given a Level 3 confidence score. Retention times were additionally predicted for Level 3 and Level 4 features where sufficient chemical information was available to facilitate molecular descriptor retrieval. The final model performance parameters, testing, training, and external validation datasets are included in **Table S5**, and the final dataset following retention time prediction and quality control measures described above is presented in **Table S6**. The final non-target dataset including confidence Levels 1, 2, and 3 is presented in **Table S7**.

Semi-quantification, Limit of Detection, Limit of Quantification. Semi-quantification was performed to estimate the concentration of tap drinking water contaminants detected and confirmed in the study. For each contaminant, calibration curves with a minimum of six calibration concentrations were constructed, and concentrations were determined from the original tap water data using the Sciex OS software and 1/x weighting. The lowest standard used for the calibration curve was analyzed in triplicate to calculate the limit of detection (LOD) as follows:

$$LOD = \frac{k * S_b}{m}$$

where k = the confidence factor 3, S_b = the standard deviation of instrument response in counts per second of the lowest standard, and m = the slope of the calibration curve line. Limits of quantification (LOQ) *were not calculated* because concentration estimates were semi-quantitative and lacked recovery correction via extraction or internal standards. Without full quantitative validation, assignment of formal quantitation limits would be unreliable. Therefore, only LODs were calculated to define detection sensitivity. All reported sample intensities were above the LOD or were marked as *<LOD* in **Table S9a** if some evidence of presence (e.g., correct retention time and parent ion *m/z*) was present but not decisively above background noise, below mass error tolerances, or above the LOD. Conservative LODs were determined to ensure minimal reporting of false detections. Final concentrations were calculated in the SciexOS software. Final reported contaminant concentrations include blank subtraction (the largest blank value between instrument, method, or field blank) and dilution adjustment. Table **S9b** displays LODs and individual calibration curves used for semi-quantification.

Zebrafish Morphology and Behavioral Screening.

Zebrafish Husbandry. Wild type (Tropical 5D) zebrafish (*Danio rerio*) were raised and maintained at Sinnhuber Aquatic Research Laboratory (SARL) at Oregon State University. Fish were reared in brood stock tanks with water recirculation systems, with the monitored temperature to ensure a stable 28 ± 1°C and a stable pH of 7.4. Fish were fed twice daily with standard lab diet (Sparos Zebrafeed 300 μm), and tank water was supplemented with Instant Ocean salts (Spectrum Brands, Blacksburg, VA, USA). The fish were spawned by placing a specialized funnel tanks the night before and at first light the next morning; spawning would initiate over the gridded

platform. The embryos are then collected and screened for unfertilized embryo or malformations prior to being sorted by developmental life stage in embryo medium (EM).[8]

Chemicals Screening. Analytical grade stock solutions of the chemicals used in this study were obtained through the Chemical Mixtures Core of the Oregon State University Superfund Research Center. Chemicals with little or no prior early life-stage zebrafish toxicity screening data were first tested individually. Zebrafish chemical exposures, photomotor behavioral assays, and toxicity screening was carried out as described in Rivera et al.[9] Briefly, zebrafish embryos were collected on the day of the exposure and transferred to a temperature-controlled incubator at 28 ± 1°C. At four and a half hours post fertilization (hpf), zebrafish embryos were dechorionated and transferred into 96-well round-bottom plates pre-filled with 100 µL EM, resulting in 12 replicate embryos for eight individual concentration points per chemical.[10] At 6.5 hpf, embryos were exposed to stock concentrations with 100% DMSO dispensed into individual wells using a HP D300e digital dispenser, and were normalized to 1% dimethyl sulfoxide (DMSO). An initial static range-finding exposure was carried out with nominal chemical concentrations of 0.00, 1.00, 2.54, 6.45, 16.40, 35.00, 74.80, and 100.00 µM. Plates were kept for 18 hours on a shaker at 225 revolutions per minute at 28°C in the dark until 28 hpf, when embryos were assessed for mortality, then kept stationary in the dark at 28°C until larvae were assessed for 120 hpf morphology. Solutions were not exchanged during the experiment.

Definitive toxicity exposures were then performed for each observed bioactive chemical, with exposure concentrations chosen individually to cover the maximum range of bioactive effects identified during the range-finding study. Three replicate plates were used for each chemical resulting in 36 replicate embryos for each concentration point and chemical. The

exposures were initiated at 6 hpf and continued to 120 hpf. An embryonic photomotor response (EPR) assay was conducted at 24 hpf to assess the activity levels of the embryos, where activity during two sequential light pulses is recorded. Abnormal behavior is identified by deviations in response between control embryos and exposed embryos indicating abnormal photomotor development. At 120 hpf, a larval photomotor response (LPR) assay was conducted, where larval movement during four sequential light and dark cycles is measured and the movement is binned into ~six second intervals. Differences between the movement in the control and exposed fish indicates photomotor developmental abnormalities in the larvae. Additionally, at 24 hpf and 120 hpf binary zebrafish mortality was evaluated. At 120 hpf, a suite of thirteen additional binary morphological endpoints were assessed by inspection under a dissecting microscope. Examples of developmental toxicity endpoints are presented in the zebrafish phenotype atlas (https://github.com/Tanguay-Lab/Bioinformatic_and_Toxicological_Resources/wiki/Zebrafish_Phenotype_Atlas).

The benchmark nominal concentration eliciting a 20% increase in response over control fish embryos or larvae (BMC_{20}) was calculated for all morphological and behavioral endpoints as described in previous work.[11,12] The 20% effect level was selected to conservatively capture responses from zebrafish, where the 10% level (i.e., BMC_{10}) was determined to be influenced considerably by background noise and random zebrafish behavior and morphology. To calculate BMC_{20} values, an unrestricted three-parameter log-logistic model was applied to the binary endpoint data to model concentration-response curves. The BMC_{20} was then derived from the modelled curves when the BMC_{20} value fell within the nominal concentration range tested. Additionally, upper and lower confidence intervals were calculated for all BMC_{20} values adapted from the method used in Morshead et al,[12] where 95% confidence intervals were used for

morphological data and 80% confidence intervals were used for behavioral data (given the increased natural variability of behavioral data). All BMC_{20} values with upper confidence intervals 40x greater than lower confidence interval values were not reported.

Sample Representative Chemical Mixtures. Following the testing of individual chemicals, exposures were conducted using mixtures of confirmed chemicals in drinking waters, based on the molar ratios of contaminants quantified in the drinking water samples. Chemical mixtures were prepared by adjusting contaminant concentrations until the most concentrated Level 1 contaminant in each sample reached a total concentration of 10 mM, whilst preserving the molar ratio to other contaminants. Two contaminants detected using the HILIC extraction protocol (quinoline-4-carboxylic acid (QCA) and N-acetyl-DL-glutamic acid (NAG)) were scaled by 1025x to account for potential concentration inflation from the lower magnification factor, and to prevent mixture composition domination. This decision was also made to extend the breadth of this survey, as neither QCA or NAG were toxic when tested individually at concentrations up to 100 µM. Starting concentrations of the mixtures are shown in **Table S10.** Upon preparation, mixtures underwent solvent exchange into DMSO. Exposures were then conducted using the same protocol used for individual chemical testing, starting with a static range-finding exposure to determine the full range of bioactive effects of each mixture. The exposures were then conducted with three replicate exposure plates across the chosen concentration range for each mixture with EPR, LPR, and morphological endpoint assays collected as described above. The BMC_{20} concentrations were calculated as done for individual chemical mixtures. Individual chemical and chemical mixture exposure BMC_{20} values with upper and lower confidence intervals and endpoint descriptions are included in **Tables S11a-c**.

Statistical Analysis. Statistical investigation including principal component analysis (PCA) and hierarchical clustering analysis (HCA) were utilized to address the major chemical compositional differences in the tap water mass spectral data. The main objectives of these analyses were to identify qualitative patterns across the data, specifically with regards to the relationship between the annotated compound profiles and public water system source water types and applied treatment protocols.

Principal Component Analysis. First, the final prioritized Level 1 and 2 annotated compound intensity profiles were imported into python and log-scaled and z-score normalized. These transformations were applied to scale the data to meet the assumptions required to use PCA, including a reasonably normal distribution, a limited number of outliers, and limited data sparsity. The data were then split into annotations derived from the SPE extractions or N_2 evap extractions to reduce bias in the magnitude of spectral features across the dataset resulting from preconcentration differences. Both scaled datasets were evaluated for normality using the Kolmogorov-Smirnov (K-S) normality test using the scipy.stats module of the Scipy package in python. Failure to reject the null hypothesis was based on *p*-values ranging between 0.20 and 0.95 and KS-statistics ranging between 0.01 and 0.25 for both datasets, indicating the distributions were acceptably normal for PCA analysis. The SPE extraction and N_2 evap extraction datasets were then analyzed with PCA facilitated by the open access software package SciKit-learn in python. Loading scores for principal components (PC's) 1 and 2 were extracted to investigate the driving factors of separation along each PC axis.

Hierarchical Clustering Analysis. Simultaneously, HCA was used to cluster the log-scaled and z-score standardized data to complement sample ordination in the PCA plots. Analysis was

completed in python using the publicly available scipy.cluster and scipy.spatial.distance modules within the Scipy package, and the sklearn.metrics module within the scikit-learn package. Dendrograms were created using 'city-block' (Manhattan) distance and 'average' linkage methods to suit the scaled chemical data. Clustering performance was evaluated using silhouette scores, the Davies-Bouldin index, and a covariance bootstrapping approach involving 1000 iterations of randomly resampled datasets to ensure cluster stability. The resulting cluster memberships for tap water samples were used to color code sample points in PCA plots in **Figure S5.**

RESULTS and DISCUSSION

Statistical Analysis: Patterns of Contaminants in Drinking Waters.

Patterns in PCA and HCA. Principal component analysis (PCA) offers a strategy to simplify patterns and sources of variation within complex datasets,[13–15] and was used here to evaluate patterns in separation between water samples based on the sample intensity profiles of Level 1 and Level 2 annotated compounds (**Figure S5**). Separate PCA biplots were generated for the N_2 evap and SPE datasets, with samples color-coded by HCA membership. The first two principal components explained over 85% of the variance in the N_2 evap dataset, and just over 63% in the SPE dataset. Initially, field blanks, method blanks, pooled sample replicates, and instrument blanks were included to assess ordination consistency, with this analysis showing generally consistent clustering (**Figure S9**). One exception is noted for principal component 2 (PC2) in the N2 evap dataset; the variation captured by PC2 is minimal (~7%) and primarily driven by instrumental drift based on the inconsistent ordination of pooled sample replicates along this axis.

Both PCA biplots showed clear separation of the commercially purified bottled water sample and the Ellmaker water sample from all other samples, consistent with HCA results where distinct clusters were formed by these two samples. Separation along PC1 in the N_2 evap dataset, which explained ~79% of the data variance, was pronounced for the bottled water and Ellmaker samples. Loading score analysis for PC1 indicated that cumulative feature intensity was a strong contributor to separation, and accordingly the cumulative mean sample intensity was highest in HCA cluster 1 including most public water systems samples, and lowest in HCA clusters 3 and 4 which included only the Ellmaker and bottled water samples, respectively. (**Figure S7**). Dendrograms and feature intensity heatmaps also show minor inter-sample variability within HCA 1, while Woodburn (HCA 2), Ellmaker, and the bottled water formed distinct clusters with higher intra-group variability (**Figure S8**). Source water type appears to influence these water chemistry characteristics, as samples in HCA 1 originated from surface or mixed-source systems, while samples in HCA 2 and HCA 3 were sourced from groundwater. These patterns suggest that hydrophilic chemical composition is significantly influenced by source water type, particularly in surface water derived drinking water samples, and treatment also contributes to separation with the highly treated bottled water separated from the other water samples.

In the SPE dataset PCA, PC1 explained 47.7% of the variance in the dataset and was most strongly correlated with the "IC0" molecular descriptor calculated for chemicals during retention time prediction. The "IC0" descriptor partially reflects diversity in structurally incorporated atoms.[16] To visualize this descriptor, we considered the ratio of the sum of heteroatoms nitrogen, oxygen, and sulfur to carbon (NOS/C) for each contaminant based on the

molecular formula assigned by MS-Dial, where more complex chemicals will have a higher ratio of heteroatoms to carbon atoms, and hydrocarbon-like or uniform chemicals will have a lower ratio. The value of the NOS/C ratio increased with decreasing and negative PC1 loading scores, suggesting enrichment of heteroatom containing compounds in HCA clusters 1 and 2 containing the Ellmaker and bottled water samples, respectively (**Figure S6a and b**). The highest PC1 loading scores were dominated by structurally related triterpenoid compounds, including madecassic acid, asiatic acid, quillaic acid, and 18β-GA (**Figure S6c**). Triterpenoid compounds are known natural substances occurring in waters and sediments of natural systems, but also have uses in medicines, agrochemicals, and as food additives (and thus, potential sources to the environment related to these uses).[17–19] In contrast, compounds with the lowest and most negative PC1 loading scores included food additives, an industrial compound, a plasticizer, and a pesticide transformation product, indicating influence of primarily synthetic contaminants. Accordingly, this axis appears to represent ordination by overwhelming influence of industrial and anthropogenic compounds (Woodburn, Ellmaker, and the bottled water) or by natural compounds potentially related to wastewater or agricultural influence (all other water samples). This interpretation is consistent with the unique ordination of the bottled water sample and the exclusive detection of BBP at one end of the axis, opposite from the Philomath and Beaverton samples with high levels of triterpenoid like compounds and detections of pesticides.

In comparison, PC2 explained 15.5% of the variance in the SPE PCA, with the ionization mode of features contributing to the ordination. High PC2 loading scores were associated with negative ionization mode, with this axis notably separating the Ellmaker sample (high PC2

ordination) from the bottled water sample (low PC2 score). Most tap water samples were distributed between these two extremes.

The PCA biplots together highlight patterns within the annotated chemical data. First, the bottled water sample was characterized by low cumulative feature intensity, high variety in atom type, and positive mode ionizing compounds. These characteristics may reflect the effects of reverse-osmosis and UV-sterilization treatments, which have been reported to preferentially remove hydrophilic compounds, allow limited passage of small cationic species, and partially oxidize natural organic matter.[20] Second, the water source type of public water systems appears to make a difference in the PCA biplot ordination, particularly in the hydrophilic portion of water chemistry captured by HILIC analysis. Conversely, hydrophobic or moderately hydrophilic compounds captured in the SPE dataset are apparently less influenced by source water type, where HCA clusters did not separate by source water type directly but grouped two surface water sourced samples with relatively high triterpenoid feature intensities: Beaverton and Philomath. The Tualatin and Mary's rivers are the source waters for the Beaverton and Philomath waters, respectively, and are the only two systems operating on surface water from the western side of the Willamette Valley in Oregon. Past work has described different compositions of triterpenoid compounds in Oregon rivers draining the Cascade Range and the Coastal Range, [18] potentially contextualizing the separation of these two samples from other surface water samples based on Cascade Range rivers. Finally, the artificial sweeteners cyclamate (CYC) and saccharin (SAC) were among the most positive PC2 loadings and were detected prominently in the Ellmaker sample. Both compounds are sulfur containing in the form of sulfonyl and sulfonic acid groups, consistent with negative mode ionization preference. In

contrast, BBP was detected in positive ionization mode and was the highest PC2 loading score in the SPE PCA, reflecting its unique presence and strong influence on the ordination of the bottled water sample.

SUPPLEMENTAL FIGURES.

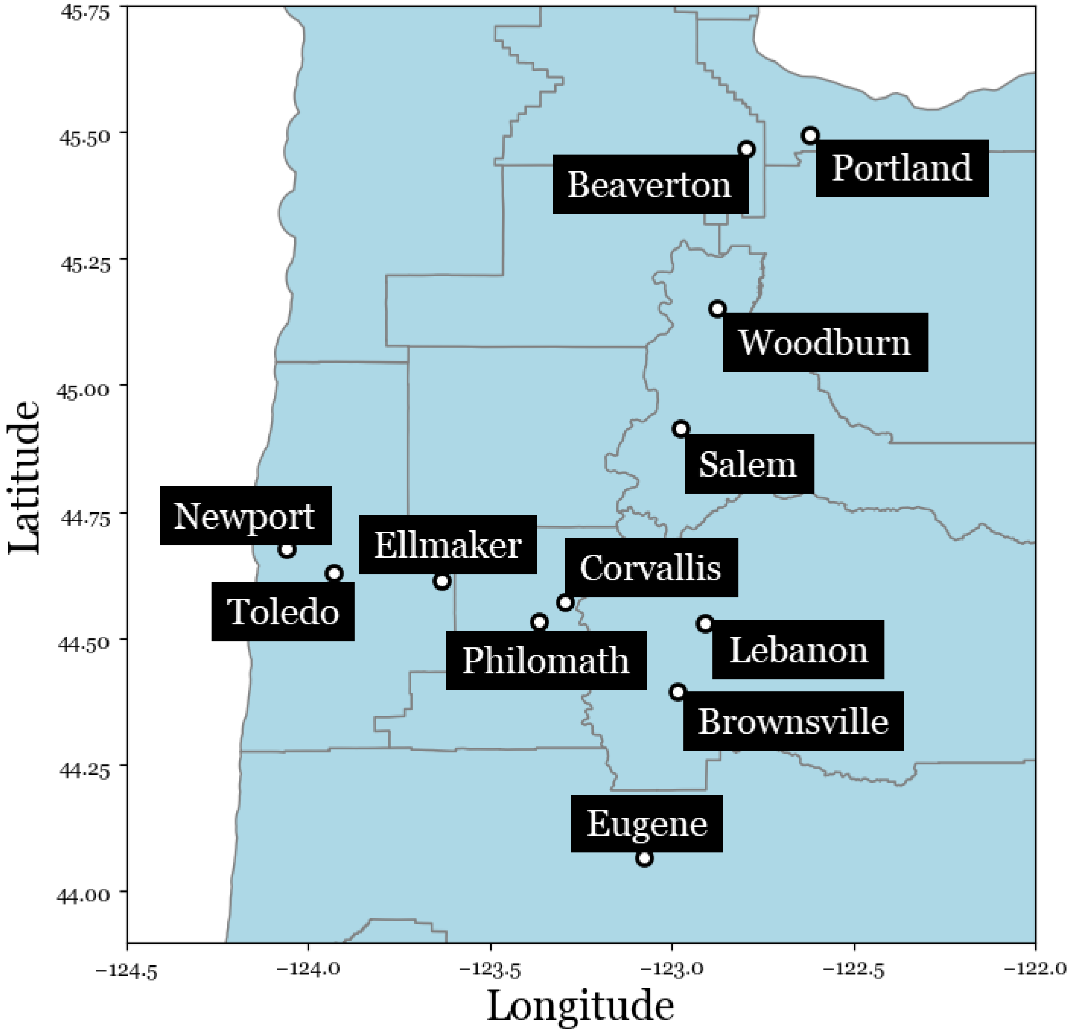

Figure S1. Water sample collection locations in Western Oregon, USA (bottled water sample not shown).

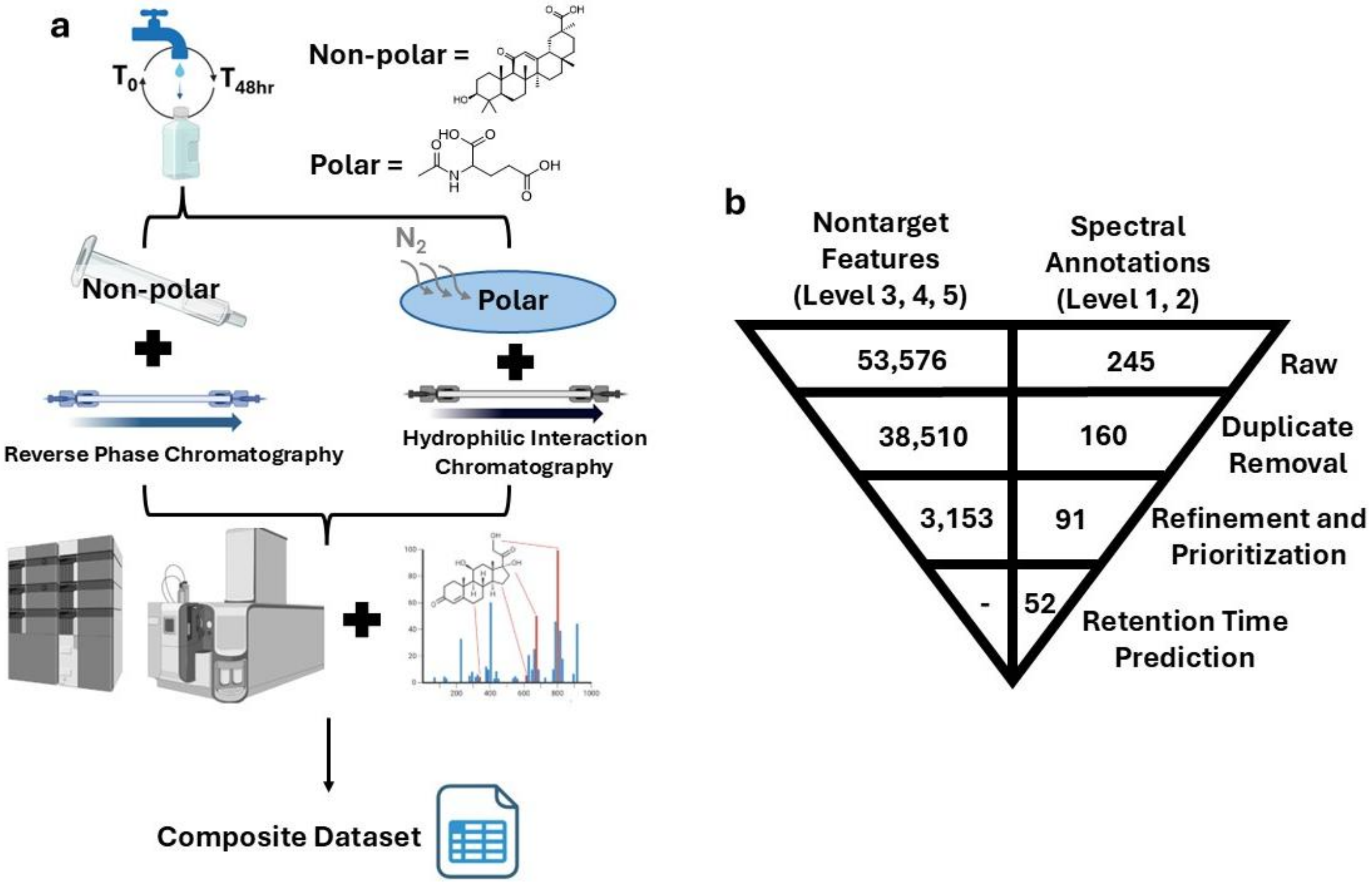

Figure S2. Overview of sample processing and data reduction workflow. Each water sample was split and extracted using two approaches to capture distinct chemical fractions: solid-phase extraction with coupled to reverse-phase chromatography for nonpolar to semipolar compounds, and evaporative extraction with nitrogen gas (N_2) coupled to hydrophilic interaction liquid chromatography (HILIC) for polar compounds (a). Extracts were analyzed by high-resolution time-of-flight mass spectrometry in positive and negative ionization modes and annotated using in-house and open-source spectral libraries. Feature counts remaining after each data reduction and prioritization step are shown in (b).

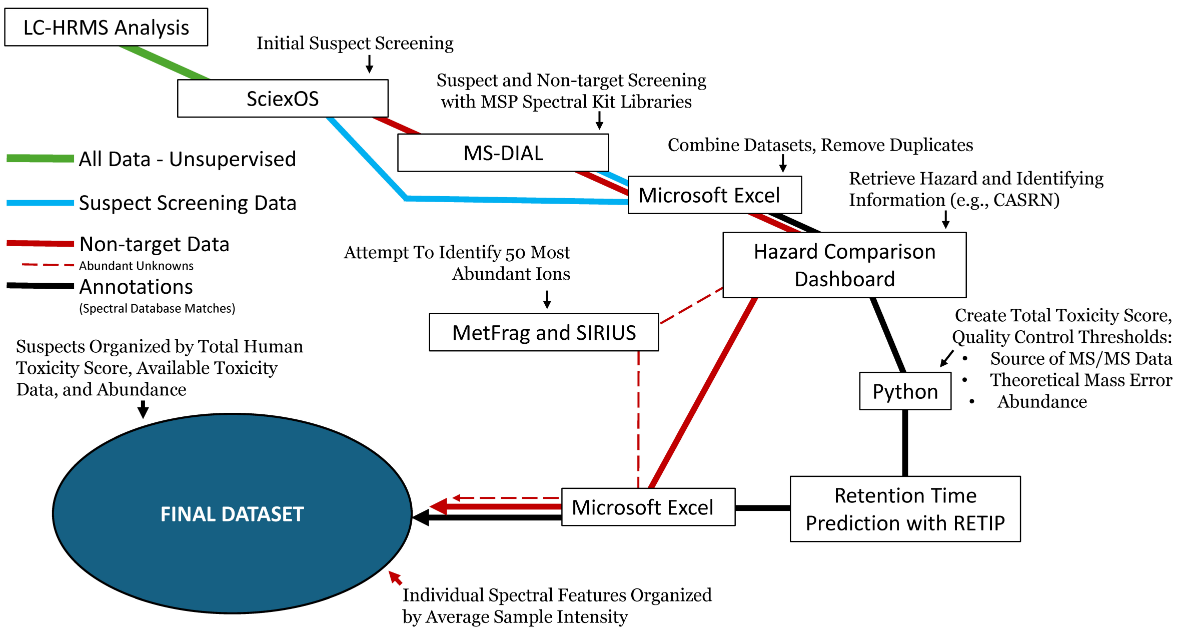

Figure S3. Schematic showing the suspect and non-target screening workflows.

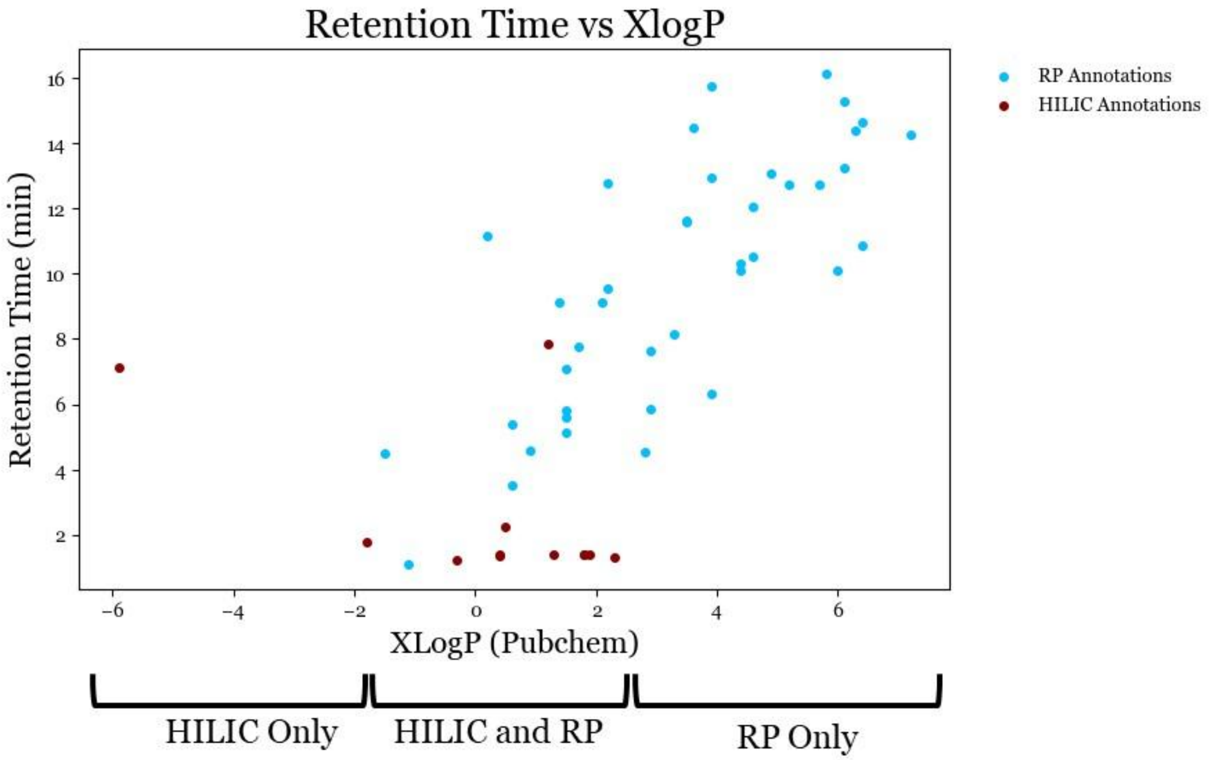

Figure S4. XlogP values were retrieved from the PubChem database for all annotated compounds. Retention time and XlogP are plotted to visualize the physicochemical range each extraction and chromatography pair isolated from drinking water.

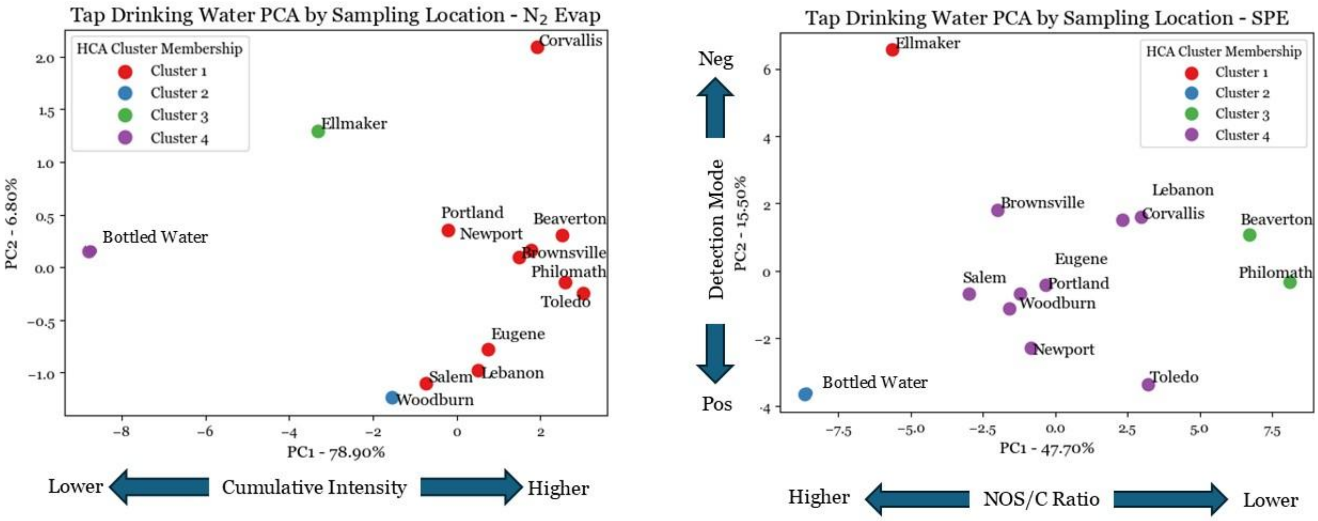

Figure S5. Individual PCA plots for principal components 1 (x-axis) and 2 (y-axis) for the level 1 and 2 annotations identified in the N_2 Evap dataset (left) and SPE dataset (right). Water samples were clustered with HCA and sample markers are color coded by their cluster membership. The PCA plot axes are labelled with proposed trends in contaminant characteristics identified by axis loading scores.

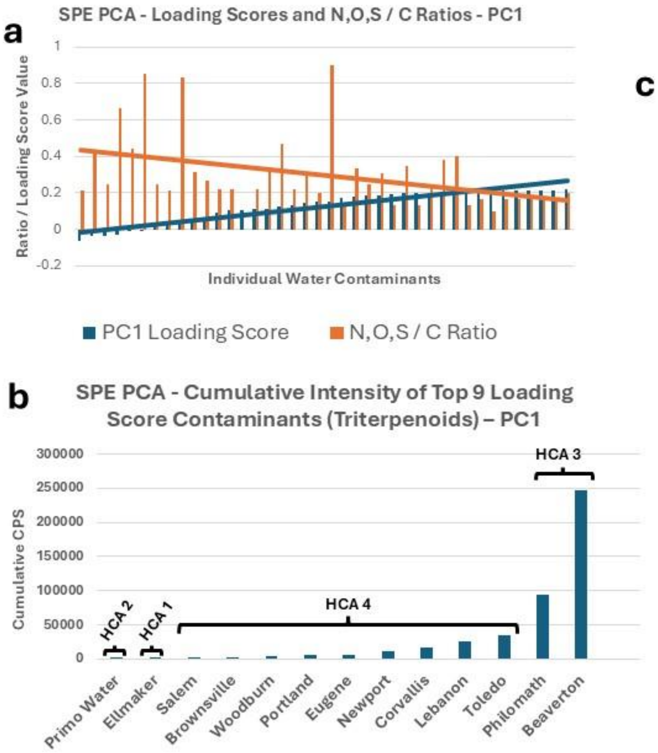

c

Key	PC1 Loading Score	INCHIKEY	Common Name
1	0.22	PRAUVHZJPXOEIF-AOLYGAPISA-N	Madecassic acid
2	0.22	JPGOJQJBPLCRQP-YHJYJCICSA-N	
3	0.22	JXSVIVRDWWRQRT-UYDOISQJSA-N	Asiatic acid
4	0.21	XJMYUPJDAFKICJ-SEAQMCRASA-N	
5	0.21	MQUFAARYGOUYEV-UAWZMHPWSA-N	Quillaic acid
6	0.21	PGQUPZCFLHXEHV-UHFFFAOYSA-N	
7	0.21	UVBLDLGZDSGCSN-MWOWFCOASA-N	
8	0.21	YNKJSQIXVXWFBK-SLGDLKFASA-N	Alisol F
9	0.21	MPDGHEJMBKOTSU-YKLVYJNSSA-N	Glycyrrhetic acid

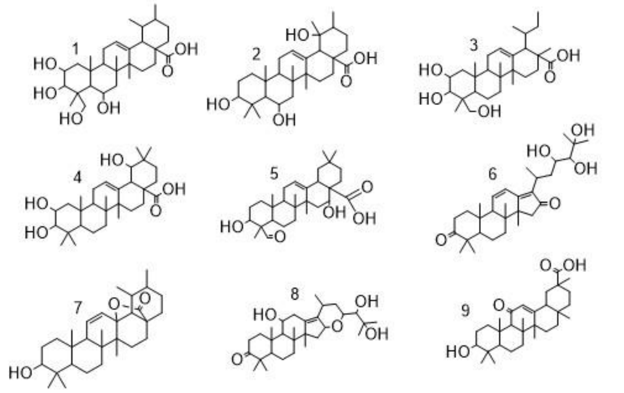

Figure S6. Evaluation of the SPE PCA loading scores; a) The NOS/C ratio, revealing lower loading scores correlated with more heteroatom rich (higher NOS/C ratio) molecules. b) A comparison of the cumulative intensities of contaminants with the top 9 PC1 loading score values. c) Individual PC1 loading scores are displayed with identifying information for each chemical with structures depicted below.

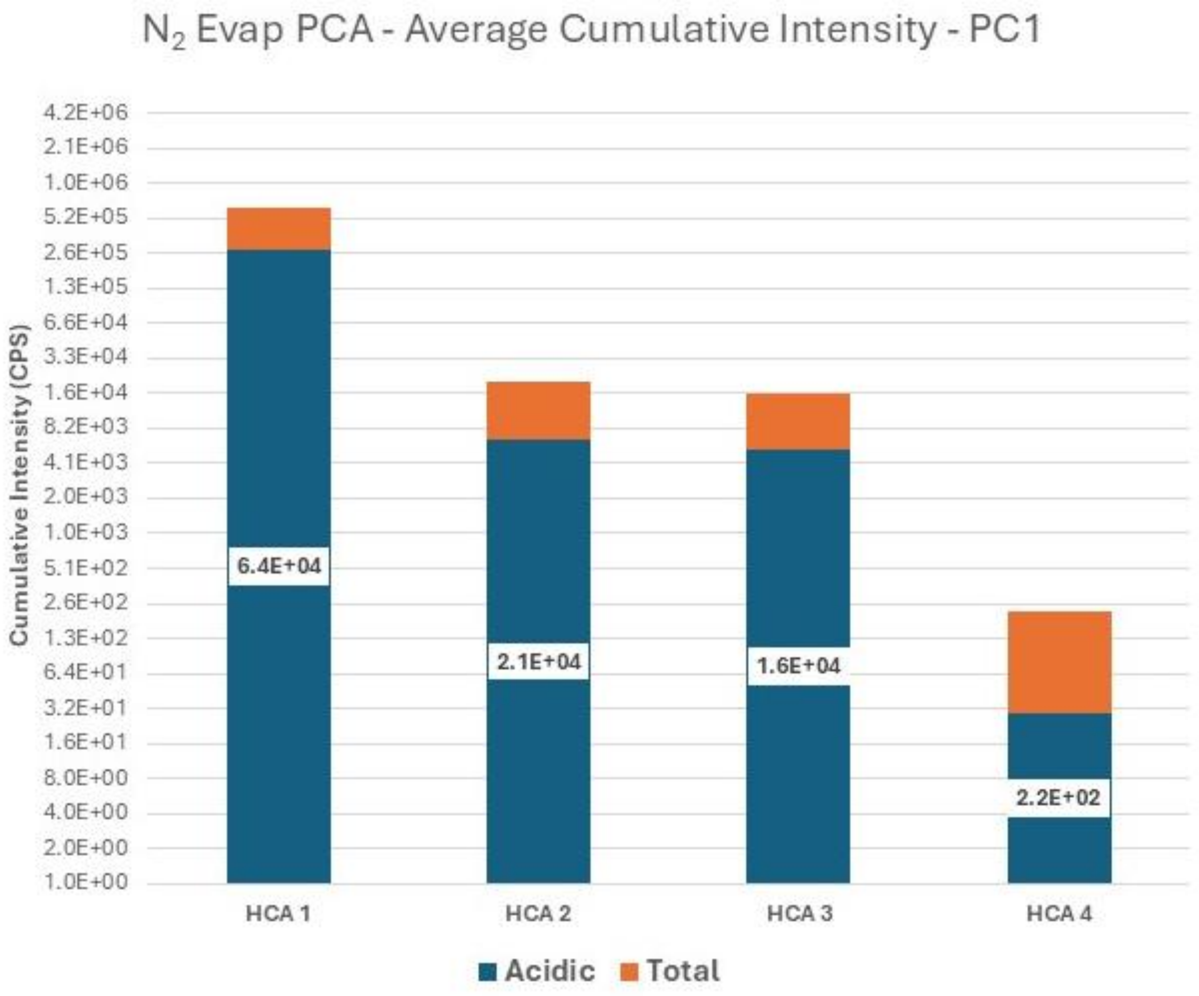

Figure S7. The N2 evap PCA revealed an association between the sample ordination along PC1 and the cumulative feature intensity and acidic feature intensity, which was also reflected in HCA cluster memberships. Histogram boxes represent the total cumulative feature intensity (blue + orange) by HCA cluster, and the subset of features containing at least one acidic moiety (blue) as defined by the RDKit molecular descriptor 'nAcid.' Numerical values within boxes indicate the cumulative intensity of acidic features in each cluster. PC2 loading scores were not evaluated due to the instability of sample ordination along PC2, as described by the inconsistent replicate pooled sample ordination in **Figure S9**.

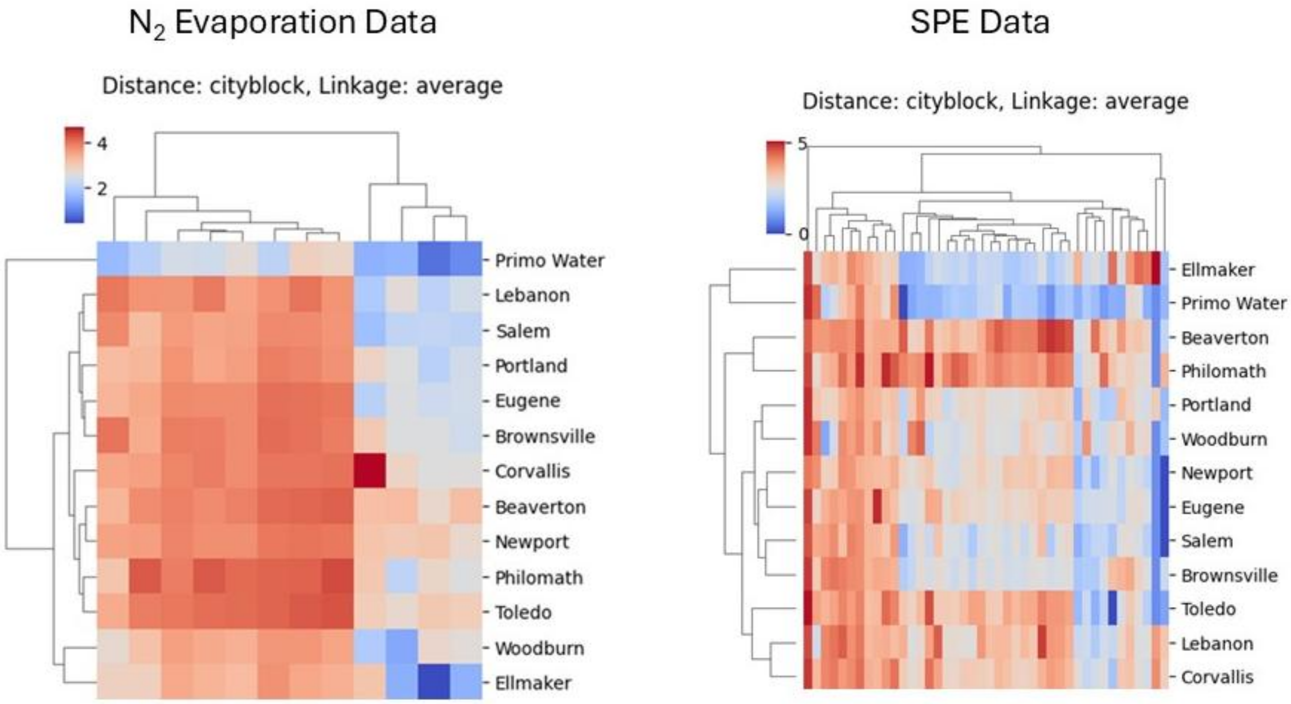

Figure S8. Hierarchical clustering analysis (city-block distance, average linkage) of contaminants included in the HCA and subsequent PCA. Heatmaps show sample (horizontal axis) and contaminant (vertical) dendrograms included in the analyses. Four clusters were selected for each dataset based on cluster stability and separation**.**

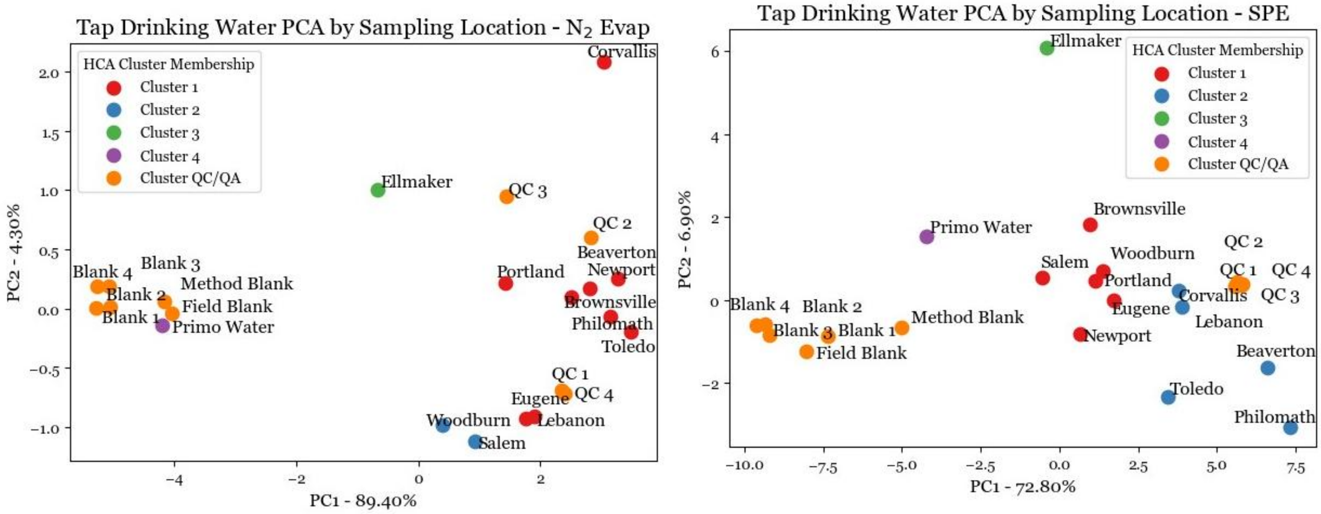

Figure S9. PCA Plots with 4 replicate instrument blanks ("Blank x"), the method blank, field blank, and pooled samples included in the analysis. In the N2 evap dataset, ordination along PC2 appears more related to instrumental drift, exceeding variance among most municipal tap water samples. PC1 captures the most variance and is the most consistent separation of the N2 evap dataset. In the SPE dataset, both PC1 and PC2 capture meaningful variation, with tight clustering of pooled and blank samples indicating meaningful separation.

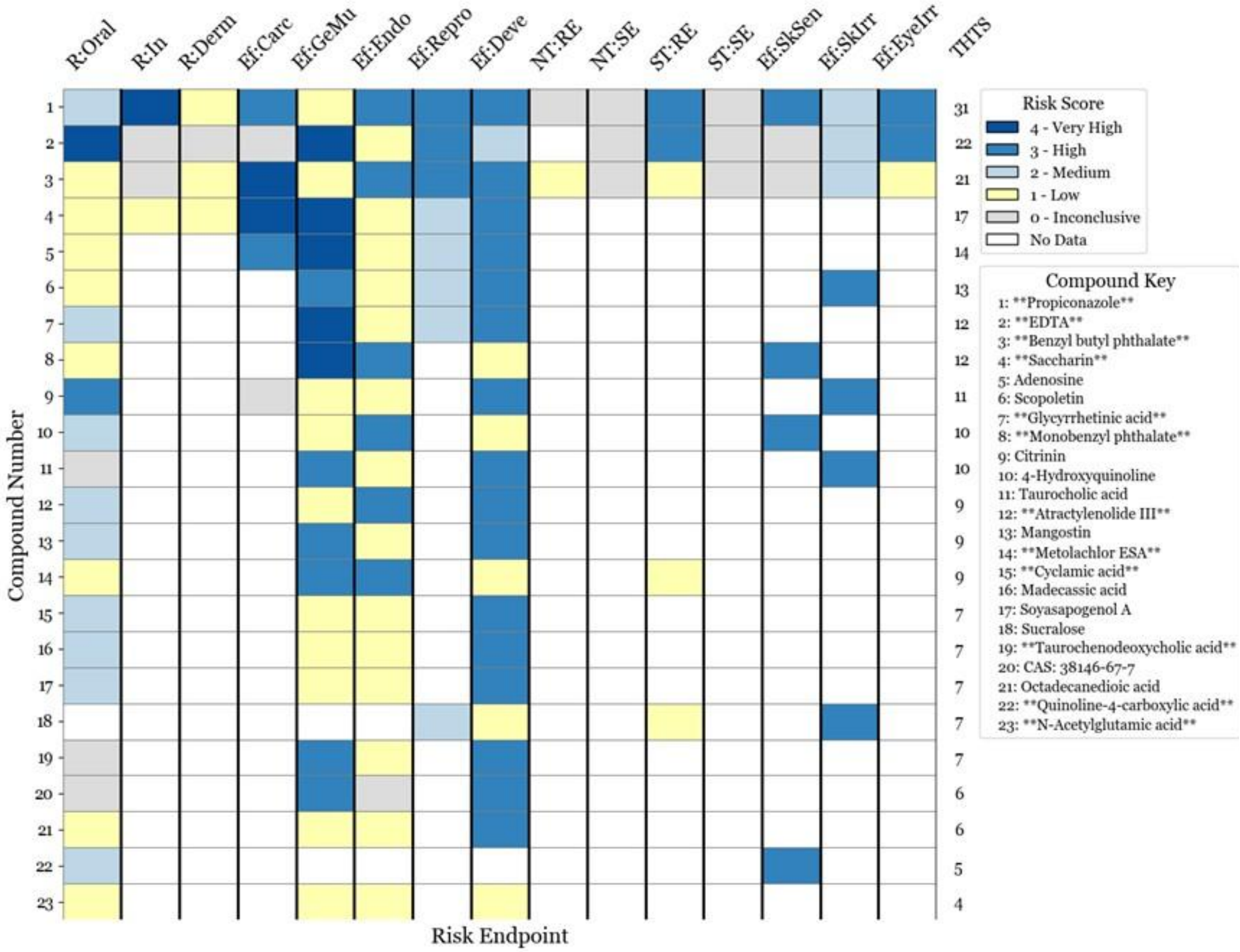

Figure S10. Hazard profiles for the 23 Level 1 and Level 2 annotations for which information was available through the USEPA's Hazard Comparison Dashboard. Chemicals marked with double asterisks (**) were confirmed at the Level 1 confidence interval. Risk endpoints are organized into four general categories: acute mammalian route of exposure risk (R:), effect endpoint (Ef:), neurotoxic endpoint (NT:), and systemic toxicity endpoint (ST:). End point abbreviations: Oral – oral risk, In – inhalation risk, Derm – dermal risk, Carc = carcinogenic toxicity, GeMu = genotoxic and mutagenic toxicity, Endo = endocrine disruption toxicity, Repro = reproductive toxicity, Deve = developmental toxicity, RE =repeat exposure risk, SE = single exposure risk, SkSen = skin sensitization risk, SkIrr = skin irritation risk, EyeIrr = eye irritation risk, and THTS = total human toxicity score. Output from the HCD given 'inconclusive' scores were given risk score values of 0 and thus did not contribute to data prioritization, however are plotted here for illustrative purposes. See **Table S11a-c** for more information about assay or score designation type.

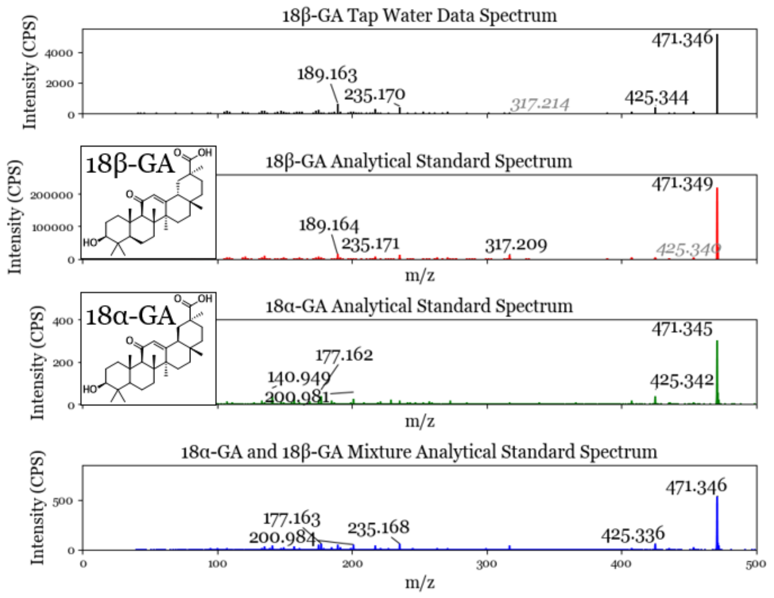

	Tap Water Data	Reference Standard Data	Tap Water Data Error
Parent Ion m/z	471.3473	471.3468	0.9 ppm*
Retention Time (min)	14.65	15.21	0.56 min

Figure S11. Structure and fragmentation data comparison of 18β-glycyrrhetinic acid, isomer 18α-glycyrrhetinic acid, and a mixture: top 5 fragment peaks in the tap water sample data (black), 18β-glycyrrhetinic acid analytical standard (red), 18α -glycyrrhetinic acid (green), and a mixture of both compounds (green) fragmentation data. *Mass error is in reference to the exact mass calculated from the compound formula within Sciex OS. Note: *m/z* value indicated in italics and gray font are not within the top 4 fragments, but are present in the spectra and specifically labelled for comparative purposes.

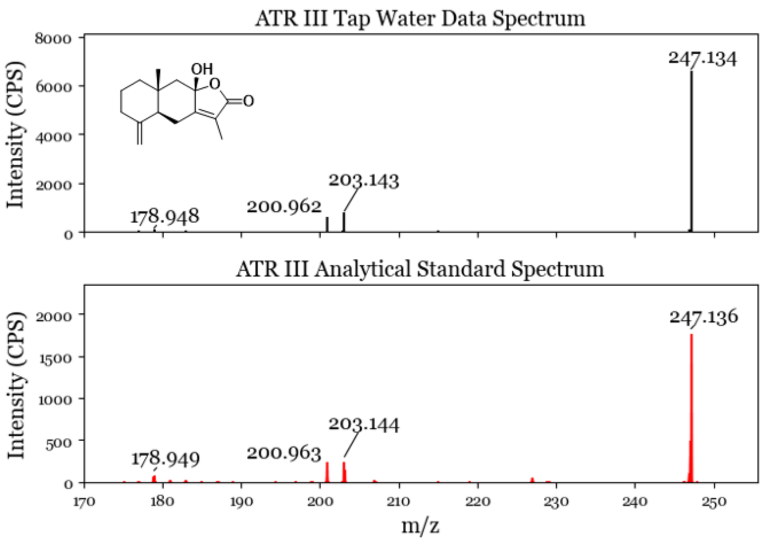

	Tap Water Data	Reference Standard Data	Tap Water Data Error
Parent Ion m/z	247.1334	247.1336	2.3 ppm*
Retention Time (min)	9.14	10.05	0.91 min

Figure S12. Atractylenolide III structure and fragmentation data comparison: top 4 fragment peaks in the tap water sample data (black) and analytical standard (red) fragmentation data. *Mass error is in reference to the exact mass calculated from the compound formula within Sciex OS.

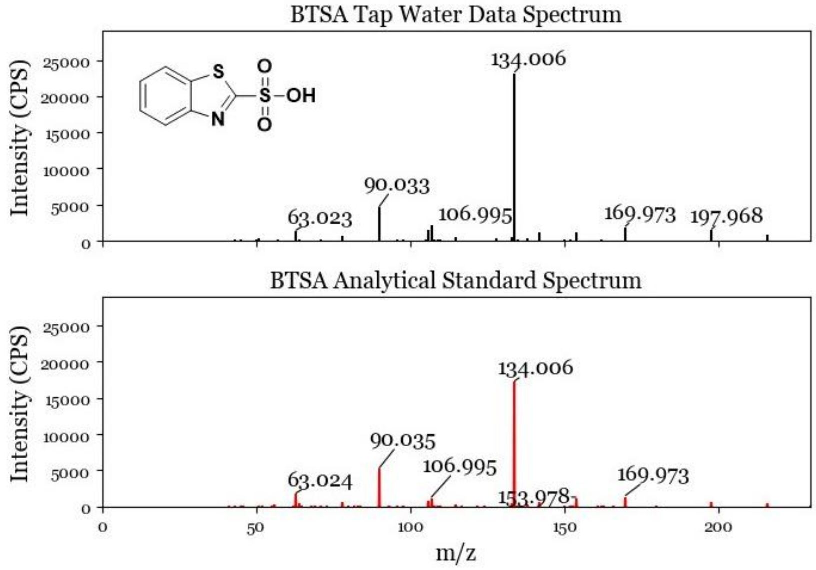

	Tap Water Data	Reference Standard Data	Tap Water Data Error
Parent Ion m/z	215.9781	215.9782	1.2 ppm*
Retention Time (min)	5.61	5.75	0.24 min

Figure S13. Benzothiazole-2-sulfonic acid structure and fragmentation data comparison: top 6 fragment peaks in the tap water sample data (black) and analytical standard (red) fragmentation data. *Mass error is in reference to the exact mass calculated from the compound formula within Sciex OS.

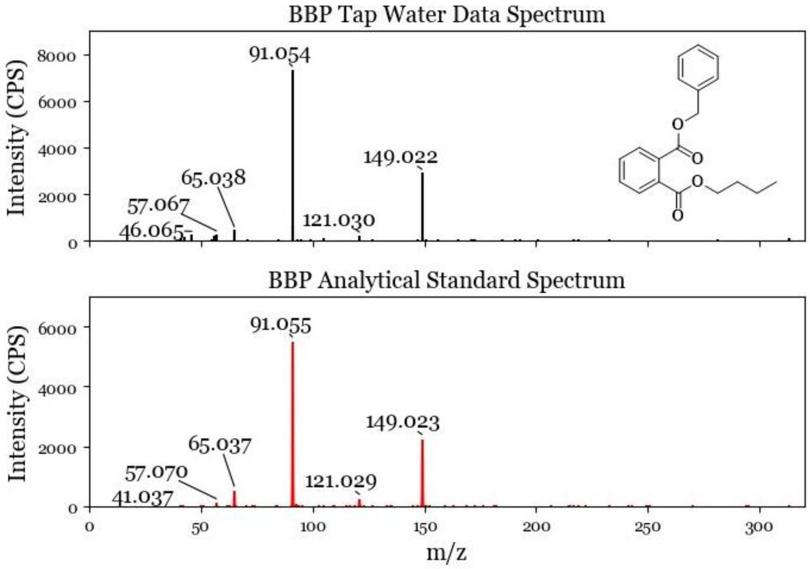

	Tap Water Data	Reference Standard Data	Tap Water Data Error
Parent Ion m/z	313.14303	313.143	1.3 ppm*
Retention Time (min)	12.85	13.03	0.18 min

Figure S14. Benzyl butyl phthalate structure and fragmentation data comparison: top 6 fragment peaks in the tap water sample data (black) and analytical standard (red) fragmentation data. *Mass error is in reference to the exact mass calculated from the compound formula within Sciex OS.

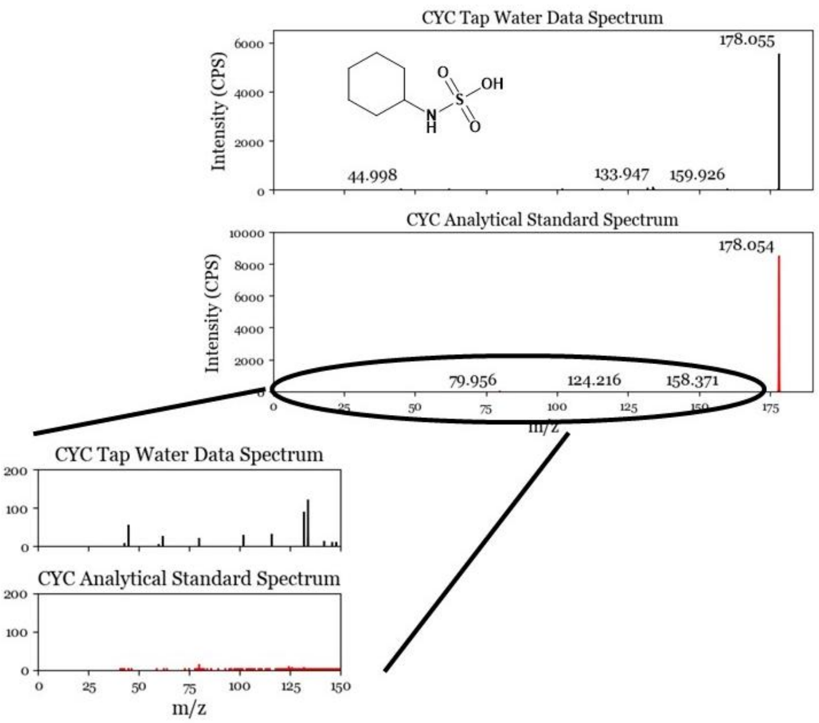

	Tap Water Data	Reference Standard Data	Tap Water Data Error
**Parent Ion m/z**	178.0541	178.0544	1.5 ppm*
**Retention Time (min)**	5.40	5.54	0.14 min

Figure S15. Cyclamate structure and fragmentation data comparison: top 4 fragment peaks in the tap water sample data (black) and analytical standard (red) fragmentation data. *Mass error is in reference to the exact mass calculated from the compound formula within Sciex OS. Note: peaks depicted in the inset were assumed to be background.

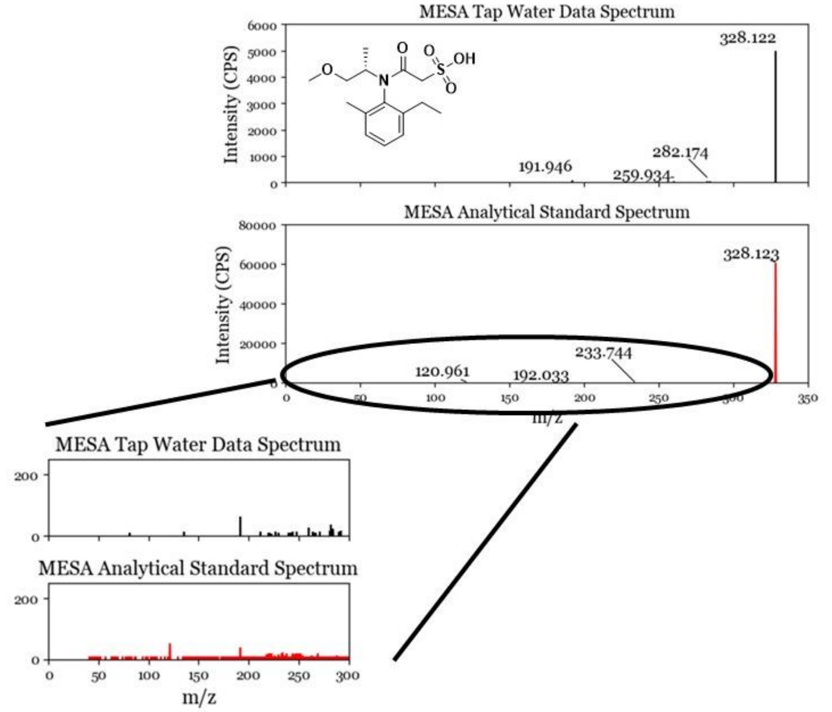

	Tap Water Data	Reference Standard Data	Tap Water Data Error
Parent Ion m/z	328.1217	328.1222	2.2 ppm*
Retention Time (min)	9.13	9.21	0.08

Figure S16. Metolachlor ethane sulfonic acid structure and fragmentation data comparison: top 4 fragment peaks in the tap water sample data (black) and analytical standard (red) fragmentation data. *Mass error is in reference to the exact mass calculated from the compound formula within Sciex OS. Note: peaks depicted in the inset were assumed to be background.

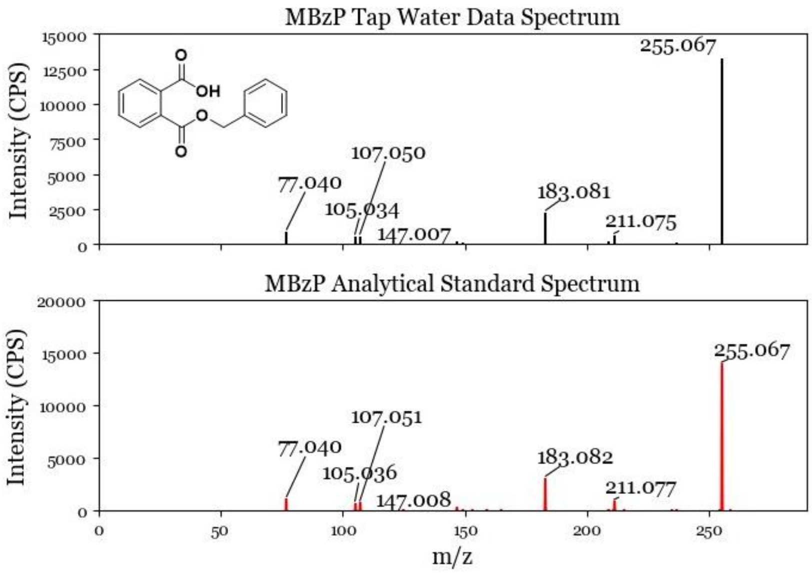

	Tap Water Data	Reference Standard Data	Tap Water Data Error
Parent Ion m/z	255.0660	255.0656	1.0 ppm*
Retention Time (min)	8.12	8.36	0.24 min

Figure S17. Monobenzyl phthalate structure and fragmentation data comparison: top 7 fragment peaks in the tap water sample data (black) and analytical standard (red) fragmentation data. *Mass error is in reference to the exact mass calculated from the compound formula within Sciex OS.

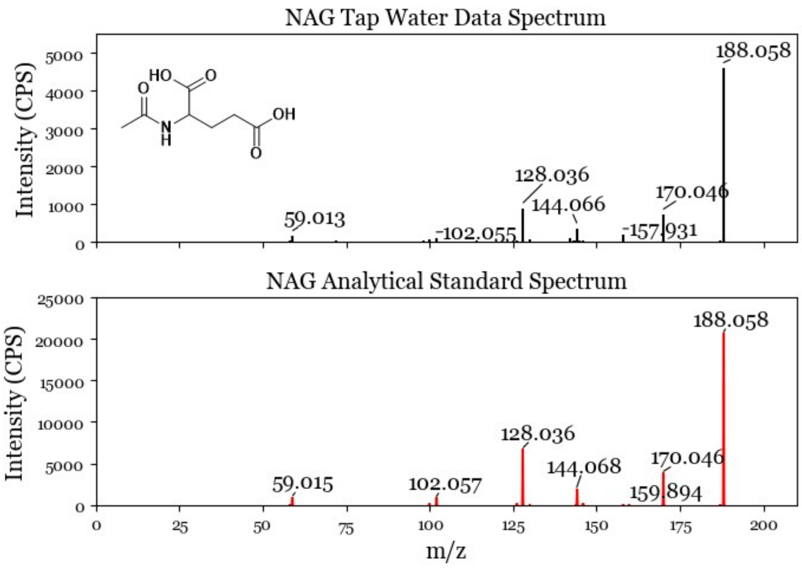

	Tap Water Data	Reference Standard Data	Tap Water Data Error
Parent Ion m/z	188.0562	188.0563	1.2 ppm*
Retention Time (min)	1.73	1.84	0.11 min

Figure S18. N-acetylglutamic acid structure and fragmentation data comparison: top 7 fragment peaks in the tap water sample data (black) and analytical standard (red) fragmentation data. *Mass error is in reference to the exact mass calculated from the compound formula within Sciex OS.

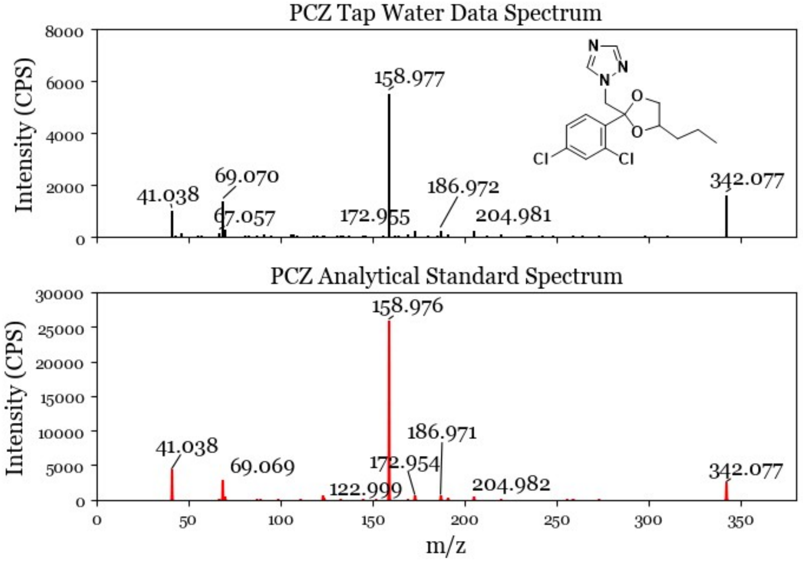

	Tap Water Data	Reference Standard Data	Tap Water Data Error
Parent Ion m/z	342.0773	342.0772	0.8 ppm*
Retention Time (min)	11.66	11.87	0.21 min

Figure S19. Propiconazole structure and fragmentation data comparison: top 8 fragment peaks in the tap water sample data (black) and analytical standard (red) fragmentation data. *Mass error is in reference to the exact mass calculated from the compound formula within Sciex OS.

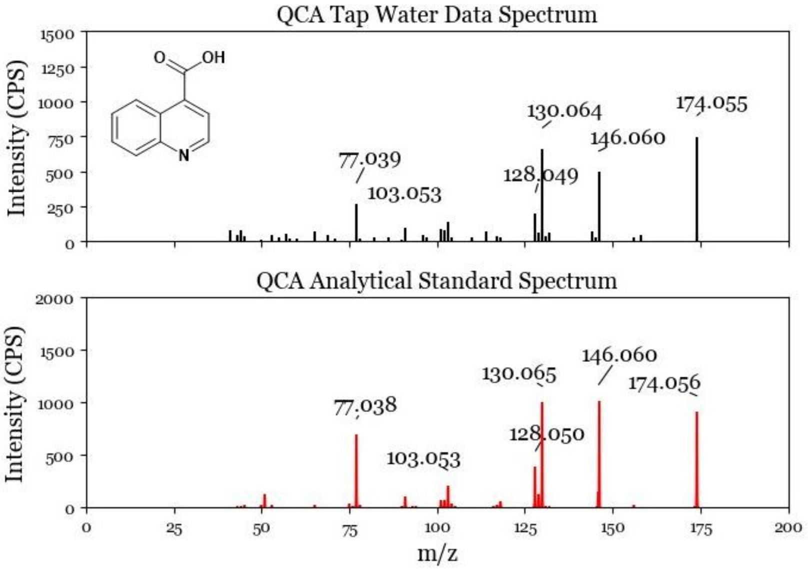

	Tap Water Data	Reference Standard Data	Tap Water Data Error
Parent Ion m/z	174.0548	174.0547	0.7 ppm*
Retention Time (min)	2.21	2.24	0.03 min

Figure S20. Quinoline-4-carboxylic acid structure and fragmentation data comparison: top 6 fragment peaks in the tap water sample data (black) and analytical standard (red) fragmentation data. *Mass error is in reference to the exact mass calculated from the compound formula within Sciex OS.

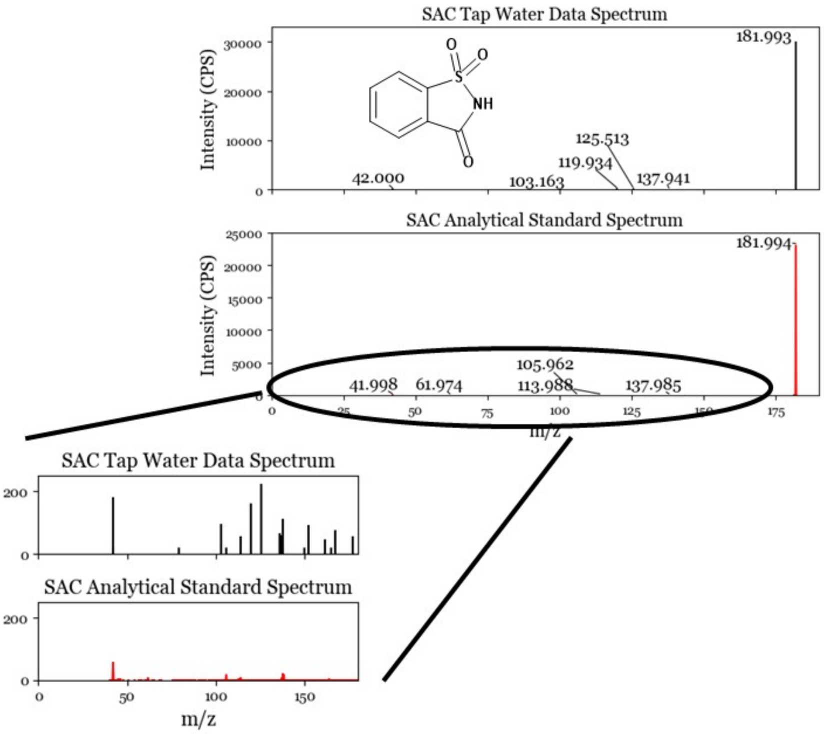

	Tap Water Data	Reference Standard Data	Tap Water Data Error
Parent Ion m/z	181.9916	181.9914	0.6 ppm*
Retention Time (min)	4.60	4.74	0.14

Figure S21. Saccharin structure and fragmentation data comparison: top 6 fragment peaks in the tap water sample data (black) and analytical standard (red) fragmentation data. *Mass error is in reference to the exact mass calculated from the compound formula within Sciex OS. Note: peaks depicted in the inset were assumed to be background.

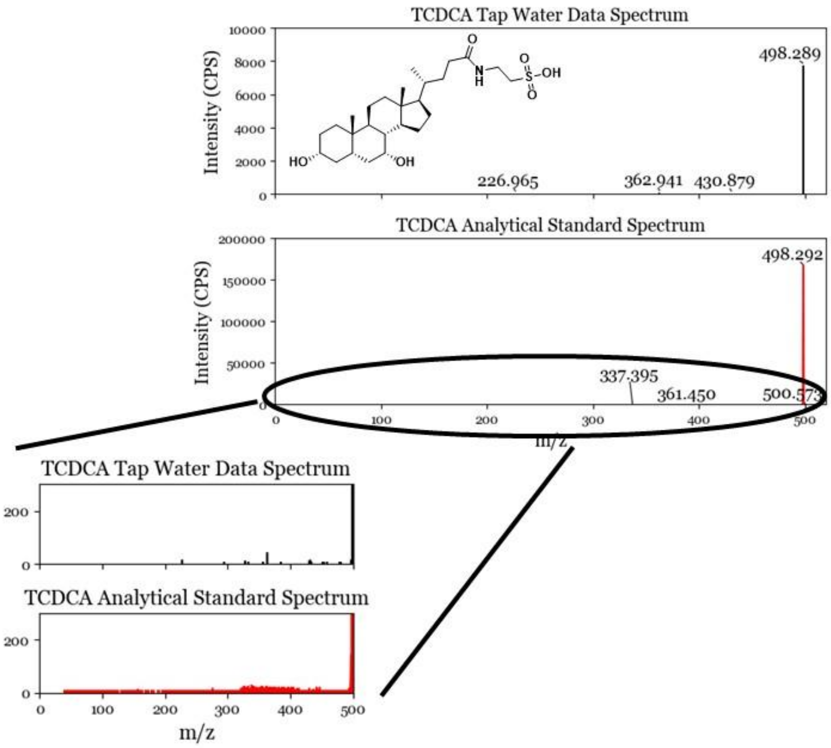

	Tap Water Data	Reference Standard Data	Tap Wate Data Erro
Parent Ion m/z	498.2883	498.2897	2.3
Retention Time (min)	14.45	14.14	0.31

Figure S22. Taurochenodeoxycholic acid structure and fragmentation data comparison: top 4 fragment peaks in the tap water sample data (black) and analytical standard (red) fragmentation data. *Mass error is in reference to the exact mass calculated from the compound formula within Sciex OS. Note: peaks depicted in the inset were assumed to be background.

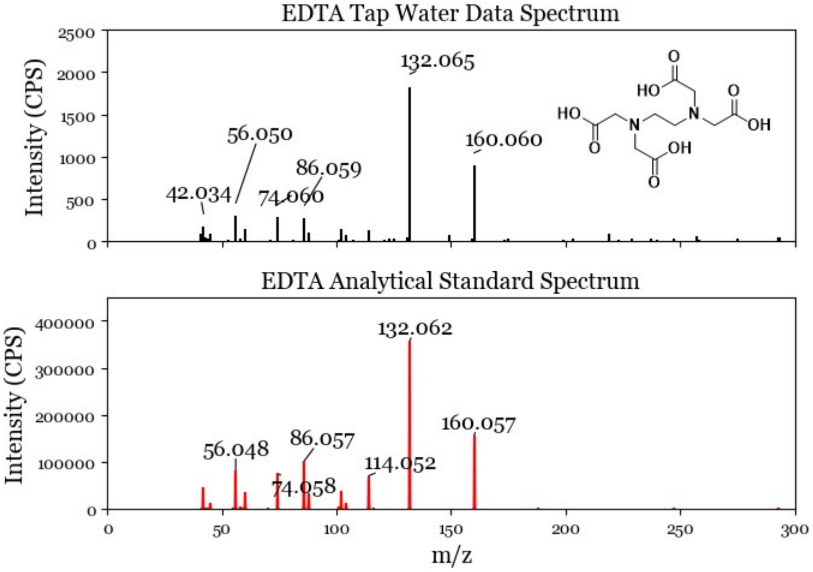

	Tap Water Data	Reference Standard Data	Tap Water Data Error
Parent Ion m/z	293.0977	293.0985	0.8 ppm*
Retention Time (min)	7.15	7.12	0.03 min

Figure S23. Ethylenediaminetetraacetic acid structure and fragmentation data comparison: top 6 fragment peaks in the tap water sample data (black) and analytical standard (red) fragmentation data. *Mass error is in reference to the exact mass calculated from the compound formula within Sciex OS.

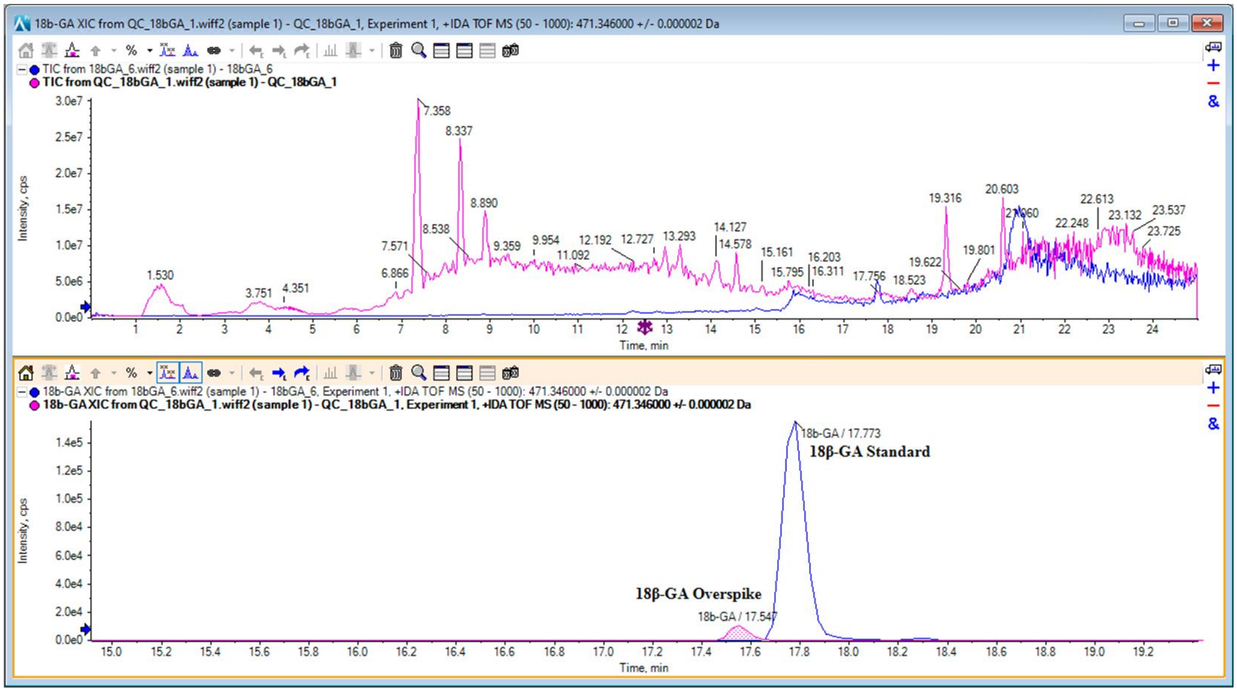

Figure S24. A retention time drift investigation of 18β-glycyrrhetinic acid (18β-GA). In pink, a standard of 18β-GA was spiked into a pooled drinking water sample extract to simulate matrix interferences ("18β-GA Overspike"), while in blue a standard of 18β-GA was prepared in pure methanol ("18β-GA Standard"). Approximately 14 seconds in retention time between the two analyses is shown, suggesting matrix effects could contribute to the 34 second difference observed between the original drinking water sample data and subsequent verification standards.

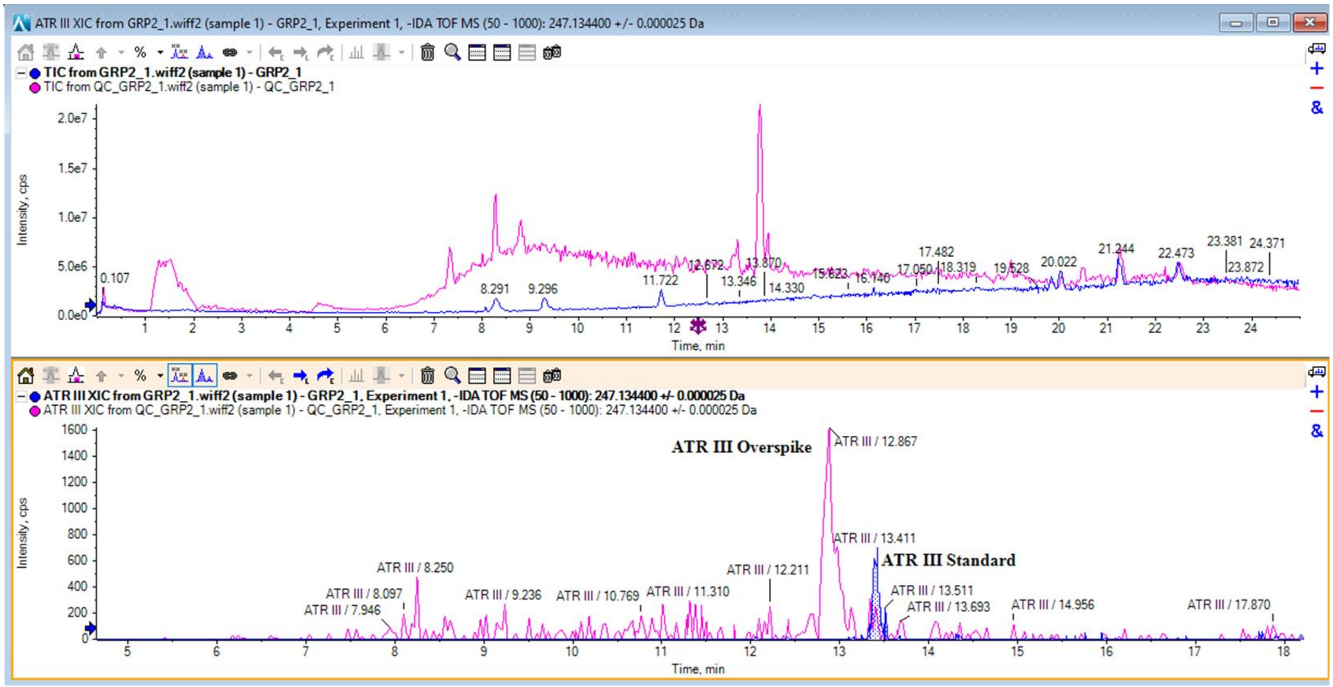

Figure S25. A retention time drift investigation of atractylenolide III (ATR III). In pink, a standard of ATR III was spiked into a pooled drinking water sample extract to simulate matrix interferences, while in blue a standard mix including ATR III was prepared in pure methanol. Approximately 33 seconds in retention time between the two analyses is shown, suggesting matrix effects could contribute to the 55 second difference observed between the original drinking water sample data and subsequent verification standards.

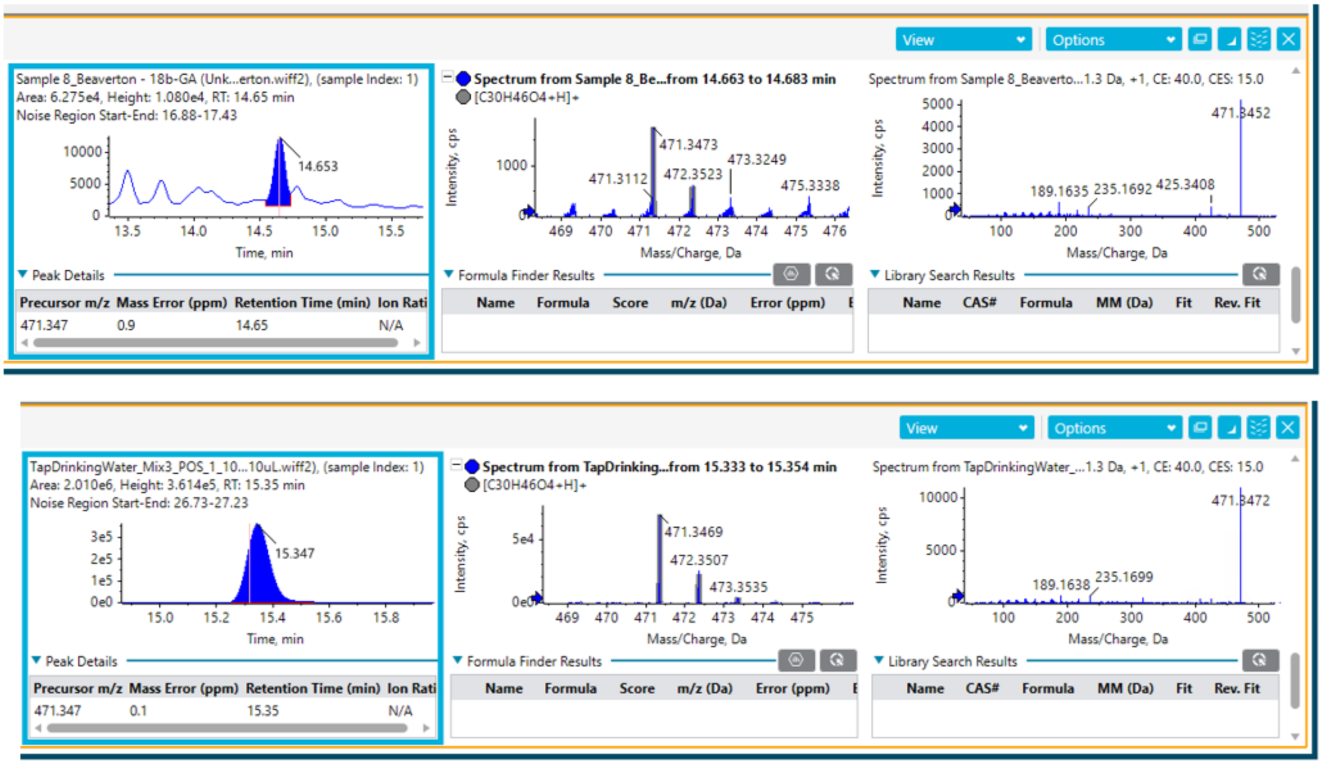

Figure S26. Chromatographic peak shape, parent ion spectra, and fragmentation spectra comparison of 18β-glycyrrhetinic acid from the original drinking water sample data (top), and subsequent verification standards (bottom).

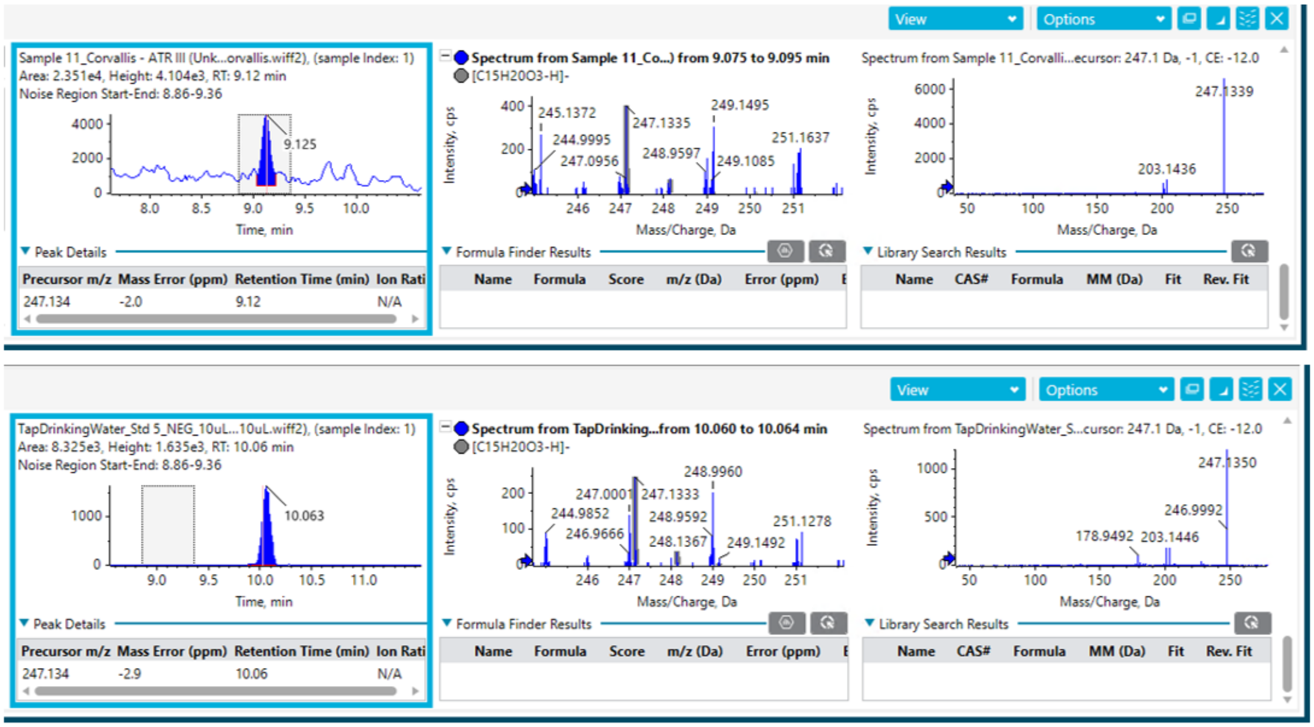

Figure S27. Chromatographic peak shape, parent ion spectra, and fragmentation spectra comparison of atractylenolide III from the original drinking water sample data (top), and subsequent verification standards (bottom).

REFERENCES

(1) Albergamo, V.; Schollée, J. E.; Schymanski, E. L.; Helmus, R.; Timmer, H.; Hollender, J.; De Voogt, P. Nontarget Screening Reveals Time Trends of Polar Micropollutants in a Riverbank Filtration System. *Environ. Sci. Technol.* **2019**, *53* (13), 7584–7594. https://doi.org/10.1021/acs.est.9b01750.

(2) Tsugawa, H.; Cajka, T.; Kind, T.; Ma, Y.; Higgins, B.; Ikeda, K.; Kanazawa, M.; Vandergheynst, J.; Fiehn, O.; Arita, M. MS-DIAL: Data-Independent MS/MS Deconvolution for Comprehensive Metabolome Analysis. *Nat. Methods* **2015**, *12* (6), 523–526. https://doi.org/10.1038/nmeth.3393.

(3) Vegosen, L.; Martin, T. M. An Automated Framework for Compiling and Integrating Chemical Hazard Data. *Clean Technol. Environ. Policy* **2020**, *22* (2), 441–458. https://doi.org/10.1007/s10098-019-01795-w.

(4) Williams, A. J.; Grulke, C. M.; Edwards, J.; McEachran, A. D.; Mansouri, K.; Baker, N. C.; Patlewicz, G.; Shah, I.; Wambaugh, J. F.; Judson, R. S.; Richard, A. M. The CompTox Chemistry Dashboard: A Community Data Resource for Environmental Chemistry. *J. Cheminform.* **2017**, *9* (1). https://doi.org/10.1186/s13321-017-0247-6.

(5) United States Environmental Protection Agency. *Provisional Peer Reviewed Toxicity Values for Butyl Benzyl Phthalate Derivation of a Carcinogenicity Assessment*; Cincinnati.

(6) Bonini, P.; Kind, T.; Tsugawa, H.; Barupal, D. K.; Fiehn, O. Retip: Retention Time Prediction for Compound Annotation in Untargeted Metabolomics. *Anal. Chem.* **2020**, *92* (11), 7515–7522. https://doi.org/10.1021/acs.analchem.9b05765.

(7) Bonini, P.; Kind, T.; Tsugawa, H.; Barupal, D.; Fiehn, O. *Retip - Retention Time Prediction for metabolomics*. https://www.retip.app/ (accessed 2026-01-03).

(8) Kimmel, C. B.; Ballard, W. W.; Kimmel, S. R.; Ullmann, B.; Schilling, T. F. Stages of Embryonic Development of the Zebrafish. *Developmental Dynamics* **1995**, *203* (3), 253–310. https://doi.org/10.1002/aja.1002030302.

(9) Rivera, B. N.; Wilson, L. B.; Kim, D. N.; Pande, P.; Anderson, K. A.; Tilton, S. C.; Tanguay, R. L. A Comparative Multi-System Approach to Characterizing Bioactivity of Commonly Occurring Chemicals. *Int. J. Environ. Res. Public Health* **2022**, *19* (7). https://doi.org/10.3390/ijerph19073829.

(10) Mandrell, D.; Truong, L.; Jephson, C.; Sarker, M. R.; Moore, A.; Lang, C.; Simonich, M. T.; Tanguay, R. L. Automated Zebrafish Chorion Removal and Single Embryo Placement:

Optimizing Throughput of Zebrafish Developmental Toxicity Screens. *J. Lab. Autom.* **2012**, *17* (1), 66–74. https://doi.org/10.1177/2211068211432197.

(11) Truong, L.; Rericha, Y.; Thunga, P.; Marvel, S.; Wallis, D.; Simonich, M. T.; Field, J. A.; Cao, D.; Reif, D. M.; Tanguay, R. L. Systematic Developmental Toxicity Assessment of a Structurally Diverse Library of PFAS in Zebrafish. *J. Hazard. Mater.* **2022**, *431*. https://doi.org/10.1016/j.jhazmat.2022.128615.

(12) Morshead, M. L.; Truong, L.; Carrell, S. J.; Scott, R.; Anderson, K. A.; Tanguay, R. L. Chemical Structure Drives Developmental Toxicity of Alkyl-Substituted Naphthalenes in Zebrafish. *Environ. Int.* **2025**, *204*. https://doi.org/10.1016/j.envint.2025.109837.

(13) Gelao, V.; Fornasaro, S.; Briguglio, S. C.; Mattiussi, M.; De Martin, S.; Astel, A. M.; Barbieri, P.; Licen, S. Self-Organizing Maps: An AI Tool for Identifying Unexpected Source Signatures in Non-Target Screening Analysis of Urban Wastewater by HPLC-HRMS. *Toxics* **2024**, *12* (2). https://doi.org/10.3390/toxics12020113.

(14) Pastore, C.; Barca, E.; Del Moro, G.; Di Iaconi, C.; Loos, M.; Singer, H. P.; Mascolo, G. Comparison of Different Types of Landfill Leachate Treatments by Employment of Nontarget Screening to Identify Residual Refractory Organics and Principal Component Analysis. *Science of the Total Environment* **2018**, *635*, 984–994. https://doi.org/10.1016/j.scitotenv.2018.04.135.

(15) Perin, M.; Dallegrave, A.; da Costa, J. S.; Streit, L.; de Araújo Gomes, A.; Pizzolato, T. M. Identification of the Organic Compounds in Surface Water: Suspect Screening Using Liquid Chromatography High-Resolution Mass Spectrometry and in Silico Toxicity Evaluation. *Int. J. Mass Spectrom.* **2023**, *484*. https://doi.org/10.1016/j.ijms.2022.116982.

(16) Basak, S.; Magnuson, V. Molecular Topology and Narcosis. A Quantitative Structure-Activity Relationship (QSAR) Study of Alcohols Using Complementary Information Content (CIC). *Arzneimittel-Forschung/Drug Research* **1983**, *33* (4), 501–503.

(17) Zakaria, F.; Norhamidah Wan Ibrahim, W.; Safinar Ismail, I.; Ahmad, H.; Manshoor, N.; Ismail, N.; Zainal, Z.; Shaari, K. LCMS/MS Metabolite Profiling and Analysis of Acute Toxicity Effect of the Ethanolic Extract of Centella Asiatica on Zebrafish Model. *Pertanika J. Sci. & Technol* **2019**, *27* (2), 985–1003.

(18) Medeiros, P. M.; Simoneit, B. R. T. Multi-Biomarker Characterization of Sedimentary Organic Carbon in Small Rivers Draining the Northwestern United States. *Org. Geochem.* **2008**, *39* (1), 52–74. https://doi.org/10.1016/j.orggeochem.2007.10.001.

(19) Luchnikova, N. A.; Grishko, V. V.; Ivshina, I. B. Biotransformation of Oleanane and Ursane Triterpenic Acids. *Molecules* **2020**, *25* (23). https://doi.org/10.3390/molecules25235526.

(20) Albergamo, V.; Blankert, B.; Cornelissen, E. R.; Hofs, B.; Knibbe, W. J.; van der Meer, W.; de Voogt, P. Removal of Polar Organic Micropollutants by Pilot-Scale Reverse Osmosis Drinking Water Treatment. *Water Res.* **2019**, *148*, 535–545. https://doi.org/10.1016/j.watres.2018.09.029.
